# Supplementary material for: Temperature-induced changes in the wheat phosphoproteome reveal temperature-regulated interconversion of phosphoforms
Source: J Exp Bot. 2018 Jun 25;69(19):4609–24. doi: 10.1093/jxb/ery204 (PMC6117581; doi:10.1093/jxb/ery204)
Supplement: Supplementary Figures [file ery204_suppl_supplementary_figures.pdf]

SUPPLEMENTARY INFORMATION

Temperature-induced changes in wheat phosphoproteome reveal temperature-regulated interconversion of phosphoforms

Lam Dai Vu[1,2,3,4,*], Tingting Zhu[1,2,3,4,*], Inge Verstraeten[1,2,§], Brigitte van de Cotte[1,2], IWGSC[5], Kris Gevaert[3,4,#], Ive De Smet[1,2,#,$]

SUPPLEMENTARY FIGURES

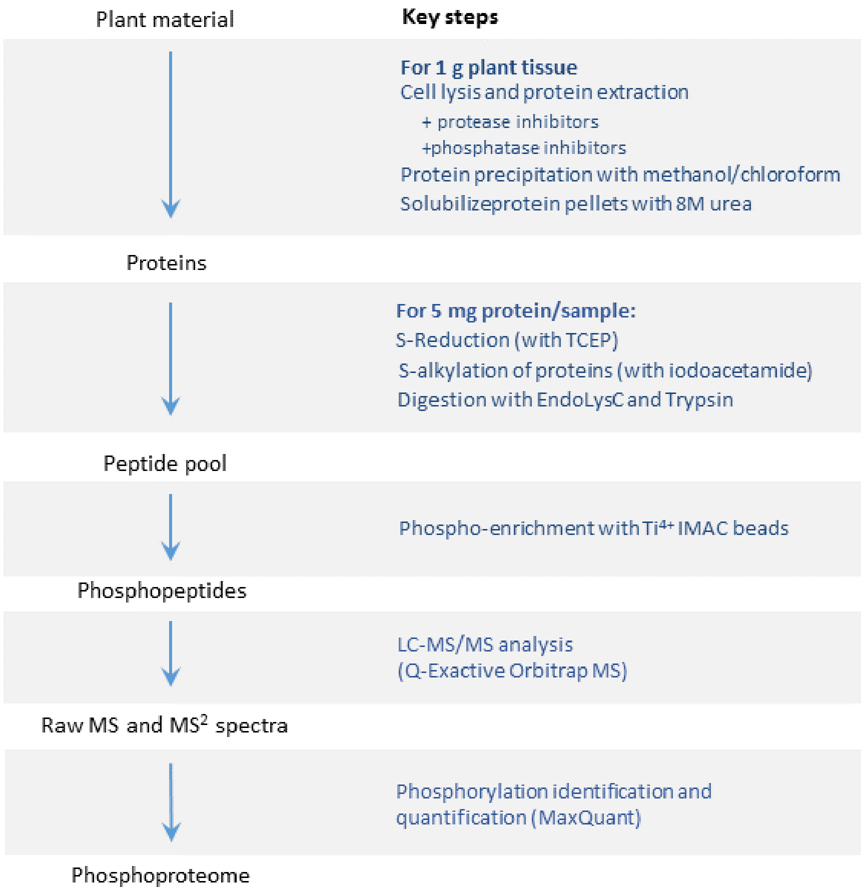

Figure S1. Summary of the phosphoproteomic workflow. The figure is adapted from Vu *et al*. (2016) *J Proteome Res* 15:4304-4317.

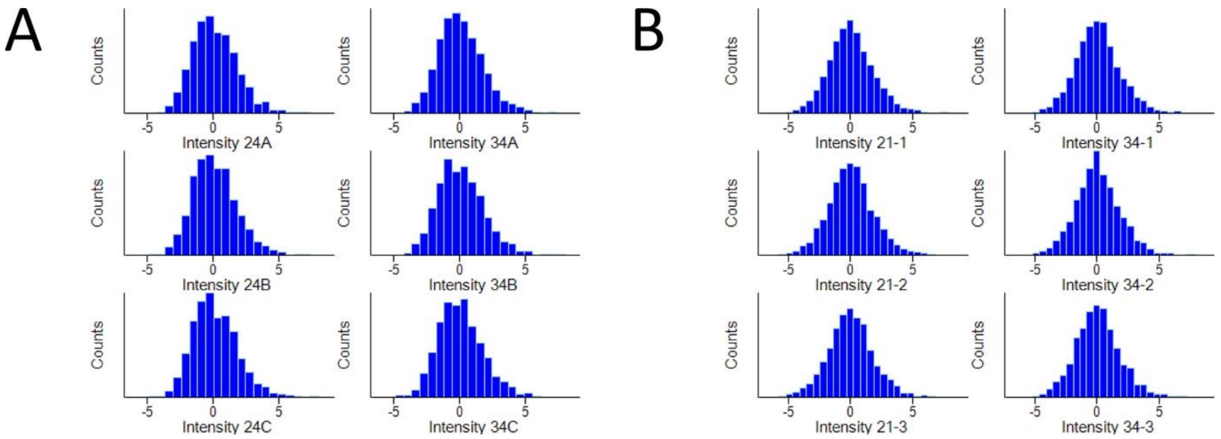

Figure S2. Histograms show normal distribution of Log2 Intensity of quantifiable proteins (proteins present in only one of two temperatures or having at least 2 valid values per temperature) in leaf (A) and spikelet (B).

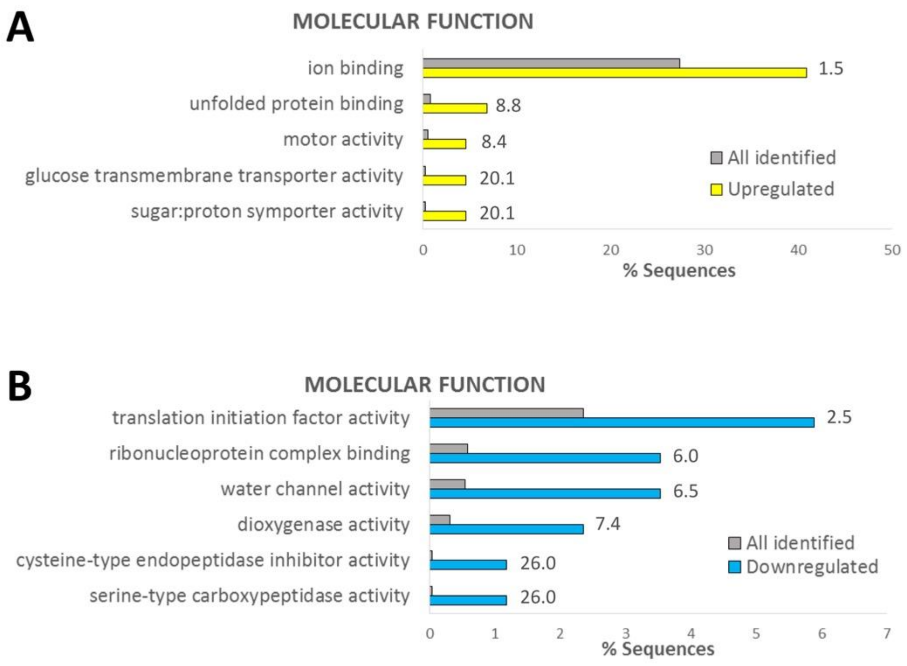

Figure S3. Overrepresented GO terms for molecular functions among leaf proteins with (A) upregulated or (B) downregulated phosphosites. Fold-changes are indicated.

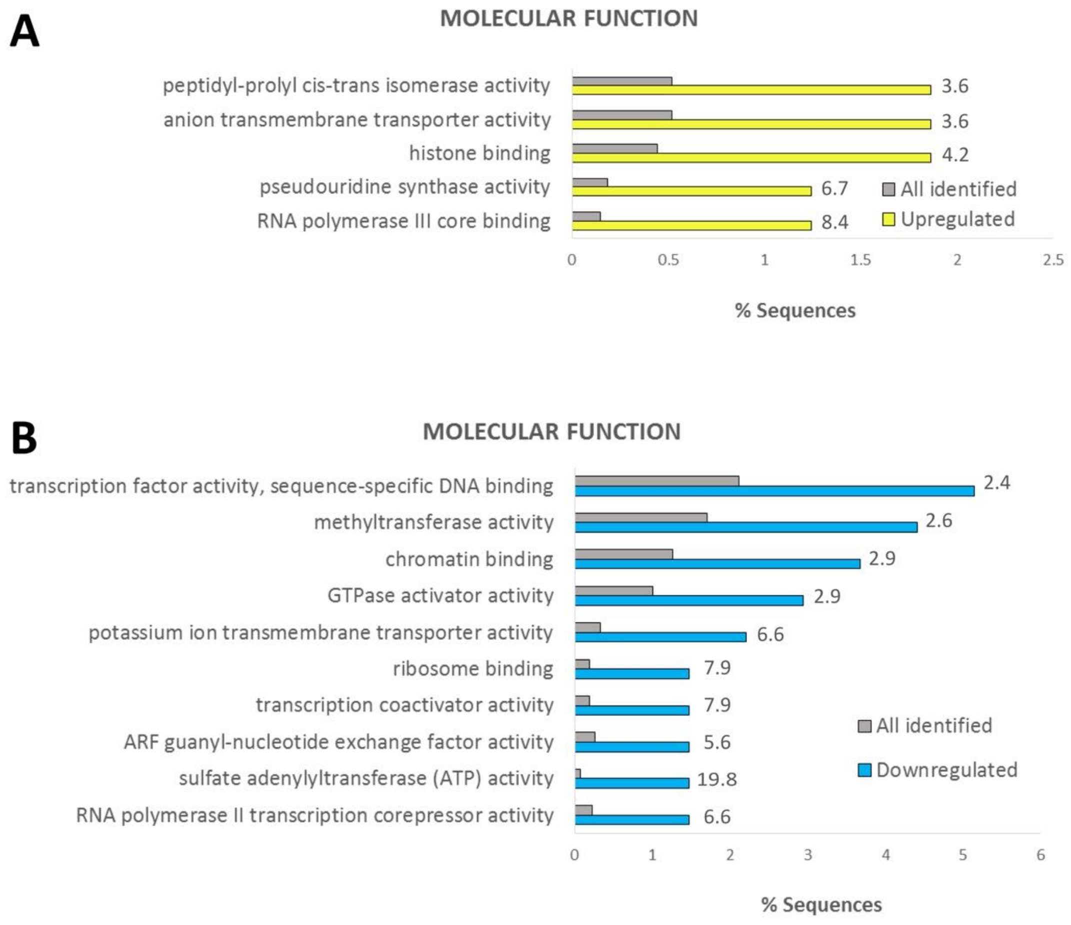

Figure S4. Overrepresented GO terms for molecular functions among spikelet proteins with (A) upregulated or (B) downregulated phosphosites. Fold-changes are indicated.

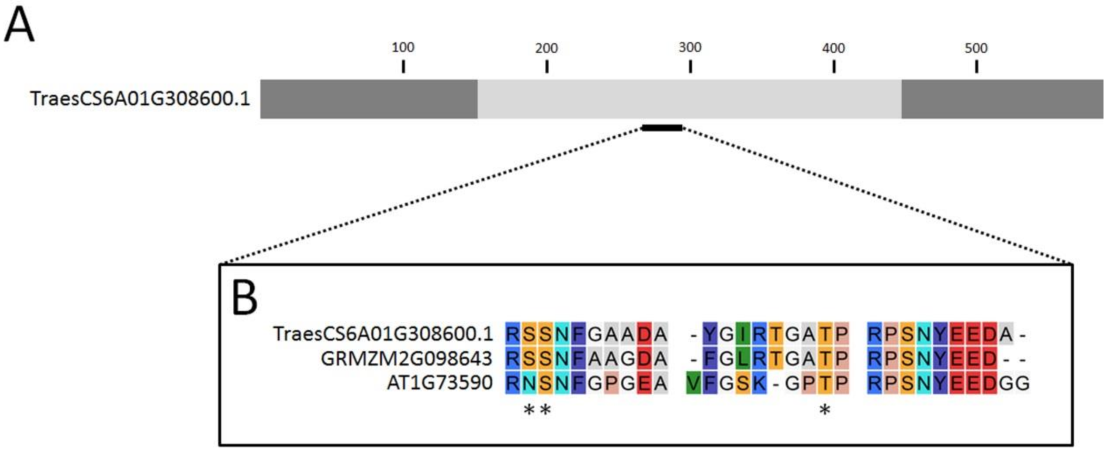

Figure S5. Conserved high temperature-regulated *Triticum aestivum* PIN1 phosphosites. **(A)** Schematic of *Triticum aestivum* PIN1 *(*TraesCS6A01G308600.1) with cytoplasmic loop in pale grey and region containing S268/S269 and T284 marked by black line. **(B)** Sequence alignment of relevant region marked in (A) in PIN1 protein sequences from *Arabidopsis thaliana* (AT1G73590), *Triticum aestivum* (TraesCS6A01G308600.1) and *Zea mays* (PIN1a, GRMZM2G098643).

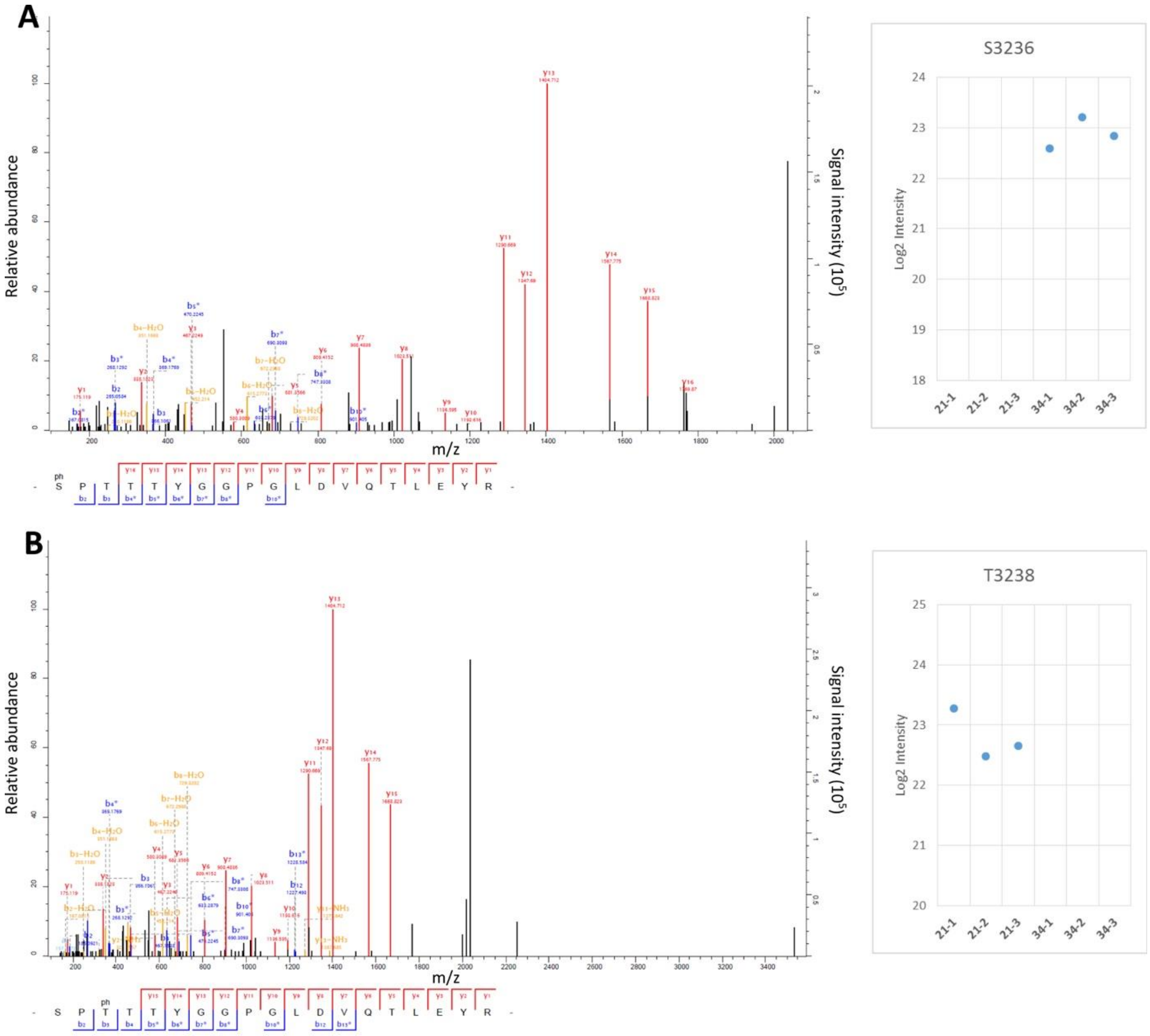

Figure S6. Mass spectrum of phosphopeptides containing S3236 (A) and T3238 (B) in SPIRRIG homologue TraesCS5B01G387800.1.

SUPPLEMENTARY TABLES

Table S1. Primers used in this study

Table S2. Phosphosites identified in wheat leaves

Table S3. Phosphosites identified in wheat spikelets

Table S4. Phosphosites uniquely present at either 24 °C or 34 °C in wheat leaves

Table S5. Phosphosites significantly deregulated at 34 °C (Students' t-test $p<0.01$) in wheat leaves

Table S6. Phosphosites uniquely present at either 21 °C or 34 °C in wheat spikelets

Table S7. Phosphosites significantly deregulated at 34 °C (Students' t-test $p<0.01$) in wheat spikelets

Table S8. Phosphosites that is commonly upregulated or downregulated at 34 °C in both leaves and spikelets

Table S9. Kinases with deregulated phosphosites in this study

Table S10. List of proteins with multiple upregulated or multiple downregulated phosphosites

SUPPLEMENTARY PROTOCOL S1

Protein Extraction and Phosphopeptide Enrichment

Total protein extraction was conducted on three biological replicate samples (leaf and flower material from independent plants) per wheat cultivar according to our previously described procedure with minor modifications (Vu *et al.*, 2017). One gram of finely ground leaf and spikelet material was suspended in homogenization buffer containing 30% sucrose, 250 mM Tris-HCl buffer (pH 8), 5 mM EDTA, and 500 mM DTT in Milli-Q water, to which the appropriate amounts of the cOmplete™ protease inhibitor mixture (Roche) and the PhosSTOP phosphatase inhibitor mixture (Roche) were added. The samples were sonicated on ice and centrifuged at 4°C for 15 min at 2500 g to remove debris. Supernatants were collected and a methanol/chloroform precipitation was carried out by adding 3, 1, and 4 volumes of methanol, chloroform and water, respectively. Samples were centrifuged for 10 min at 5000 g and the aqueous phase was removed. After addition of four volumes of methanol, the proteins were pelleted via centrifugation for 10 min at 2500 g. Pellets were washed with 80% acetone, centrifuged for 10 min at room temperature at 2500 g. The supernatants were discarded and the pellets were let to dry on air. Protein pellets were then re-suspended in 8 M ureum in 50 mM triethylammonium bicarbonate (TEAB) buffer (pH 8). Alkylation of cysteines was carried out by adding tris(carboxyethyl)phosphine (TCEP, Pierce) and iodoacetamide (Sigma-Aldrich) to final concentrations of 15 mM and 30 mM, respectively, and the samples were incubated for 15 min at 30°C in the dark. Five mg of protein material was pre-digested with MS-grade lysyl endopeptidase (Wako Chemicals) for 4 h at 37°C at an enzyme-to-substrate ratio of 1:500 (w:w),. The mixtures were diluted 8-fold with 50 mM TEAB, followed by an overnight digestion with trypsin (Promega) with an enzyme-to-substrate ratio of 1:100. The digest was acidified to pH 3 with trifluoroacetic acid (TFA) and desalted using SampliQ C18 SPE cartridges (Agilent) according to the manufacturer’s guidelines. For phosphopeptide

enrichment, the desalted peptides were fully dried in a vacuum centrifuge and then re-suspended in 500 μl of loading solvent [80% (v/v) acetonitrile, 5% (v/v) TFA].

Phosphopeptide Enrichment

The enrichment for the phosphopeptide procedure was performed as reported previously (Vu *et al.*, 2017). The re-suspended peptides were incubated with 1 mg MagReSyn® Ti-IMAC microspheres for 20 min at room temperature with continuous mixing. The microspheres were washed once with wash solvent 1 (60% acetonitrile, 1% TFA, 200 mM NaCl) and twice with wash solvent 2 (60% acetonitrile, 1% TFA). The bound phosphopeptides were eluted with three volumes (80 μl) of elution buffer (40% acetonitrile, 5% NH_4OH), immediately followed by acidification to pH 3 using 100% formic acid. Prior to MS analysis, the samples were vacuum dried and re-dissolved in 50 μl of 2% (v/v) acetonitrile and 0.1% (v/v) TFA.

Database searching

MS/MS spectra were searched against the unpublished IWGSC RefSeq v1.0 database for *Triticum aestivum* (137052 entries) (https://www.wheatgenome.org/News2/Annotation-RefSeq-v1.0-URGI) with the MaxQuant software (version 1.5.4.1). For comparison, a second search against the earlier version of IWGSC popseq PGSB/MIPS v2.2 database (100344 entries), downloaded from wheatproteome.org, was performed. A precursor mass tolerance set to 20 ppm for the first search (used for non-linear mass re-calibration) and to 4.5 ppm for the main search. Trypsin was selected as enzyme setting. Cleavages between lysine/arginine-proline residues and up to two missed cleavages were allowed. S-carbamidomethylation of cysteine residues was selected as a fixed modification and oxidation of methionine residues was selected as a variable modification. The false discovery rate for peptide and protein identifications was set to 1%, and the minimum peptide length was set to 7. The minimum

score threshold for both modified and unmodified peptides was set to 30. The MaxLFQ algorithm allowing for label-free quantification (Cox *et al.*, 2014) and the ‘matching between runs’ feature were enabled. For calculation of protein ratios, both unique and razor peptides (non-unique peptides that are assigned to a protein group with the largest number of identified peptides) were selected.

SUPPLEMENTARY DATASET

Representative MS/MS spectra of discussed phosphopeptides

Raw File	Scan	Method	Score	m/z
E14714_1p_EXT-708_PSB_Inge_Ta_24C	23831	FTMS; HCD	65.22	957.94

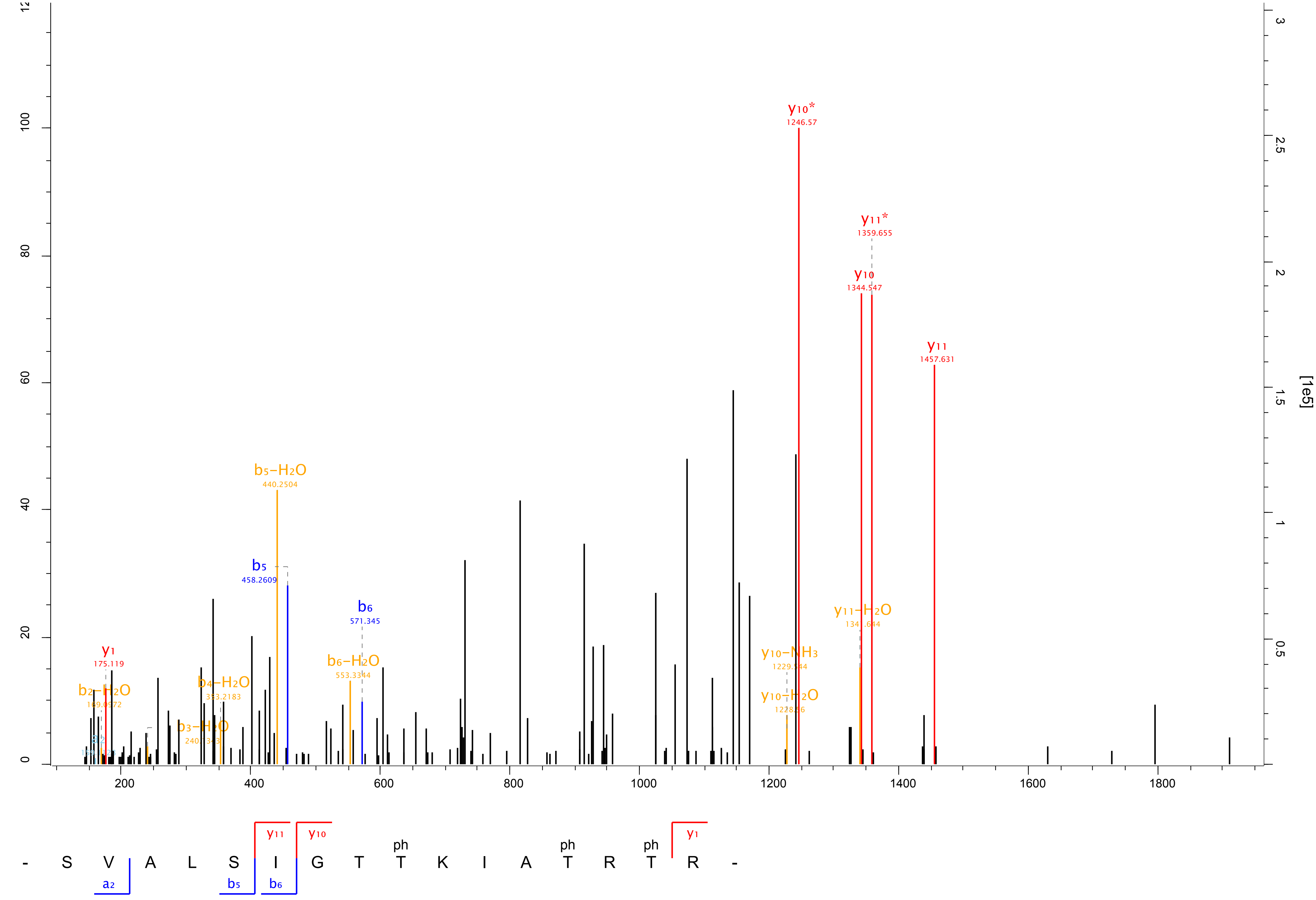

TraesCS2A01G235000.1 - T33 & T37 & T39

Raw File	Scan	Method	Score	m/z
E14710_1p_EXT-708_PSB_Inge_Ta_24A	13609	FTMS; HCD	78.53	675.77

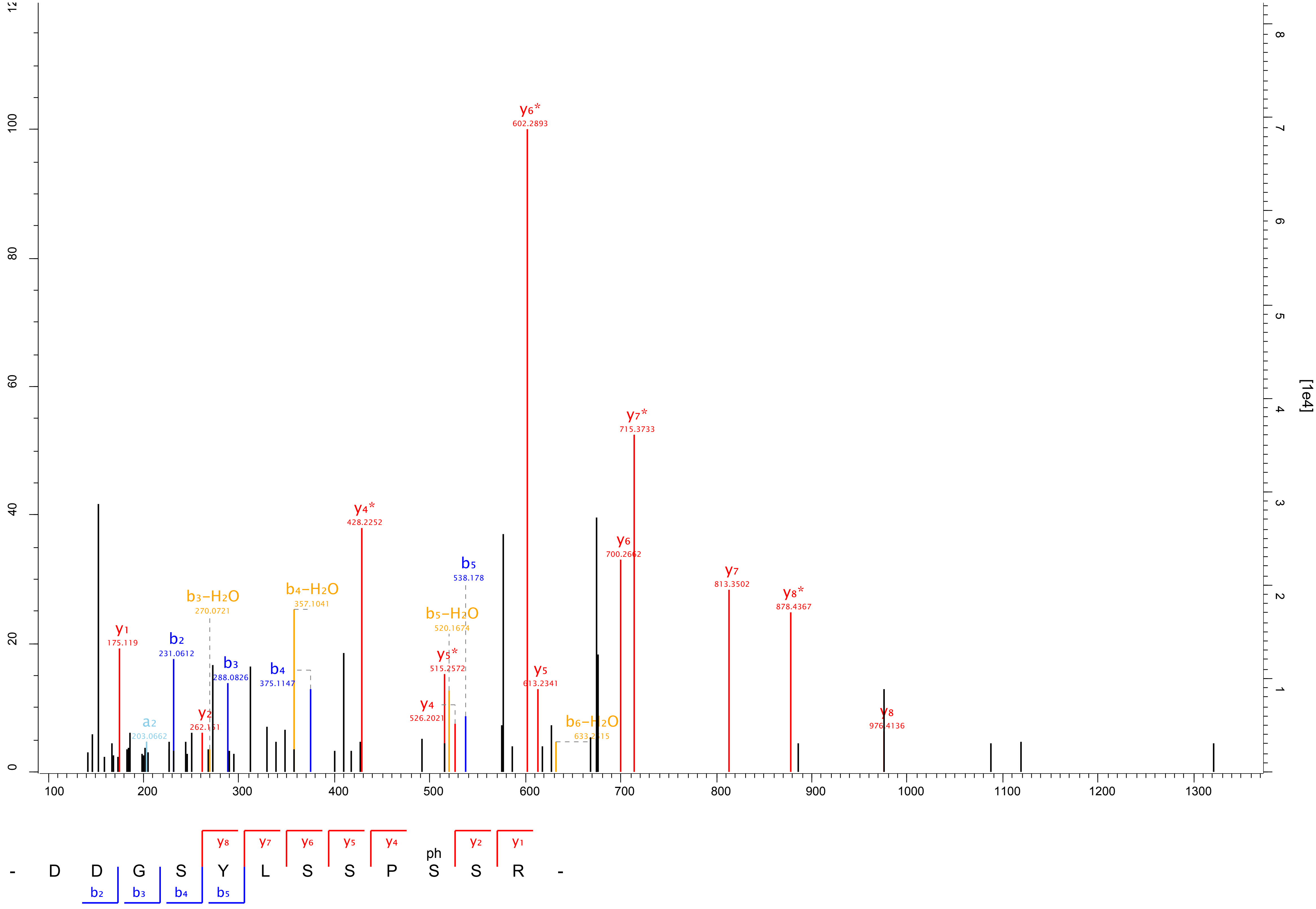

TraesCS1D01G422700.2 - S157

Raw File	Scan	Method	Score	m/z
E14716_1p_EXT-708_PSB_Inge_Ta_34A	25917	FTMS; HCD	68.54	581.78

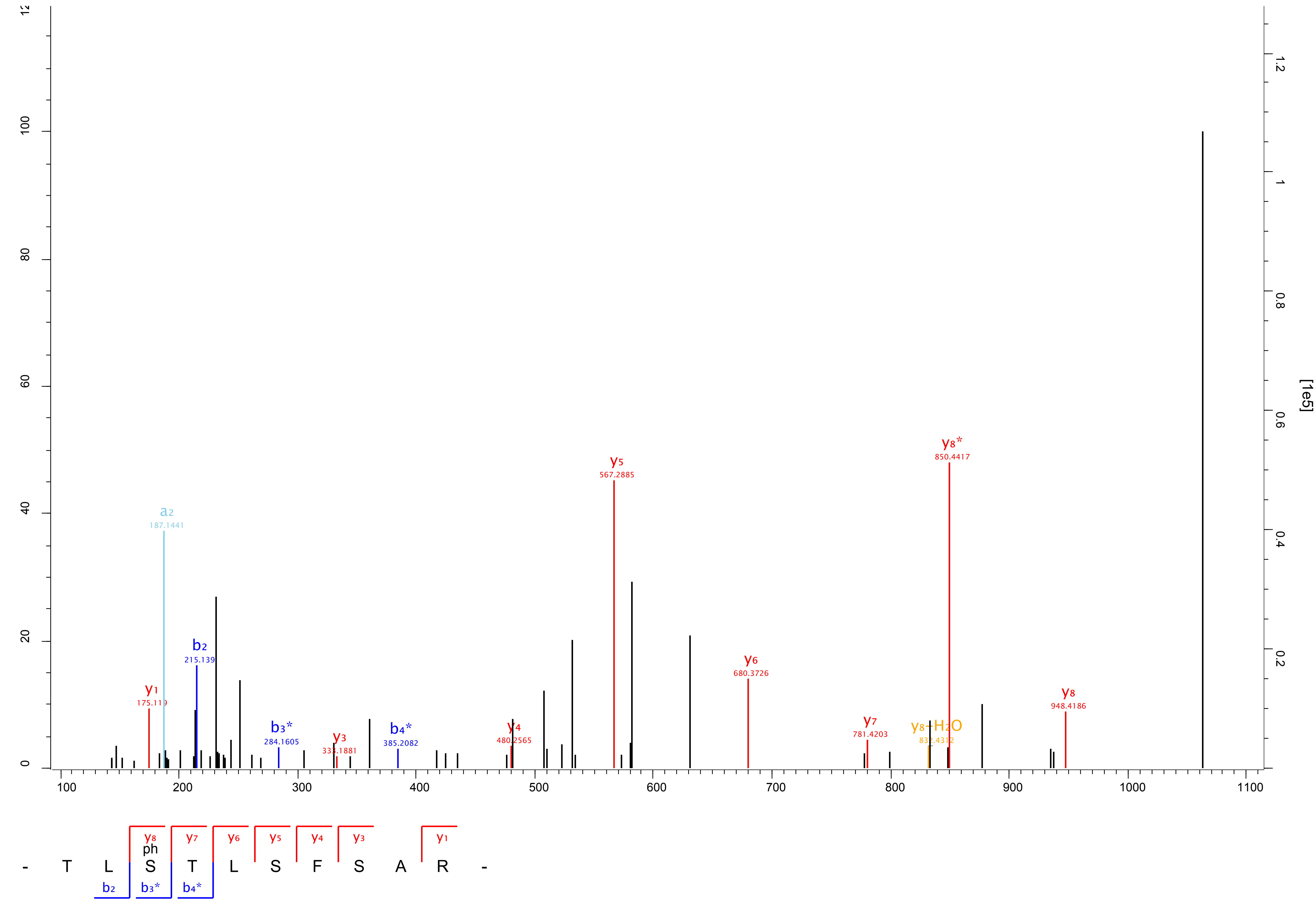

TraesCS7D01G176200.1 - S444

Raw File	Scan	Method	Score	m/z
E14716_1p_EXT-708_PSB_Inge_Ta_34A	22613	FTMS; HCD	157.38	719.87

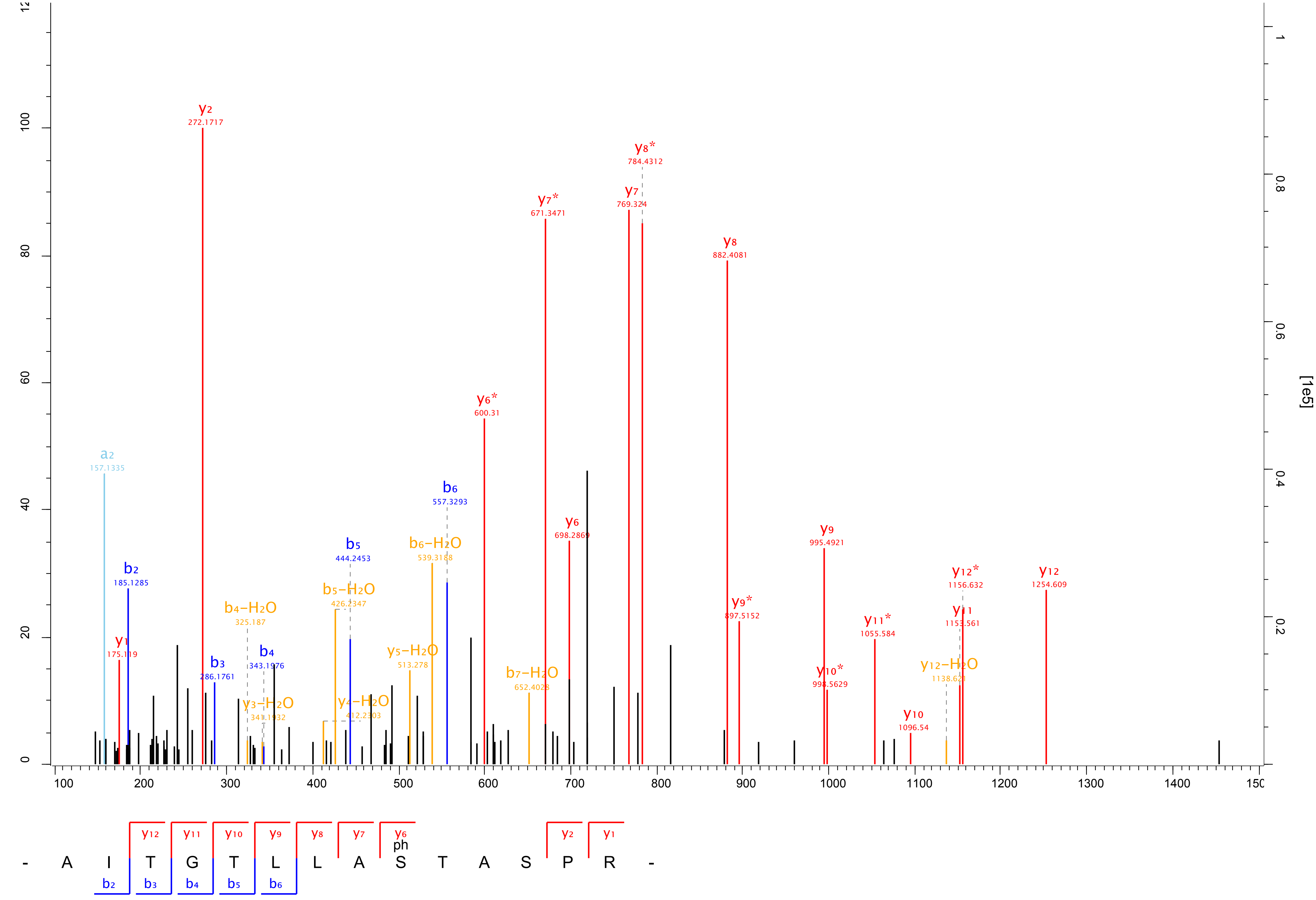

TraesCS5D01G389200.2 - S525

Raw File	Scan	Method	Score	m/z
E14714_1p_EXT-708_PSB_Inge_Ta_24C	24923	FTMS; HCD	66.62	494.71

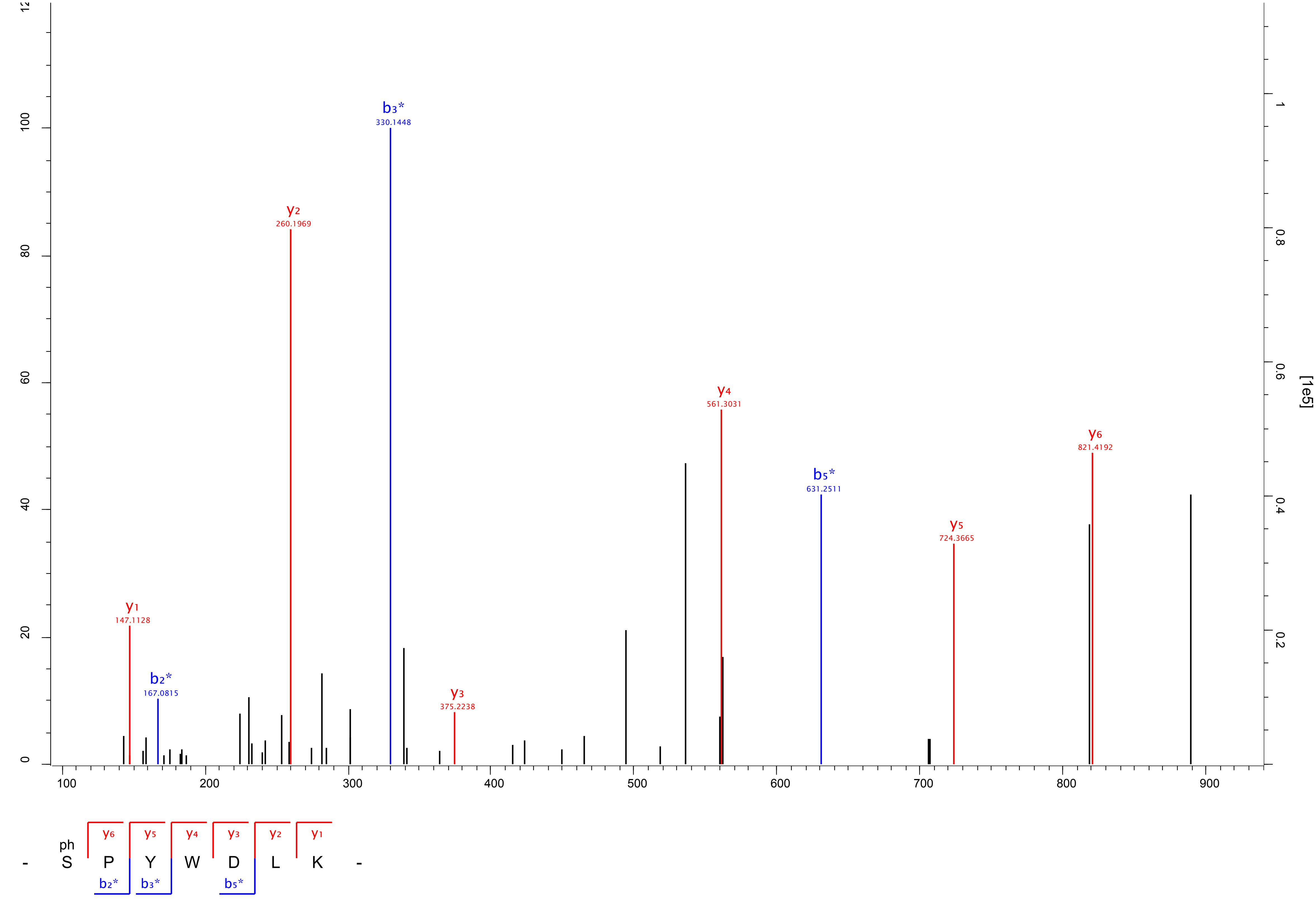

TraesCS2B01G290500.3 - S294

Raw File	Scan	Method	Score	m/z
E14716_1p_EXT-708_PSB_Inge_Ta_34A	11306	FTMS; HCD	60.76	865.39

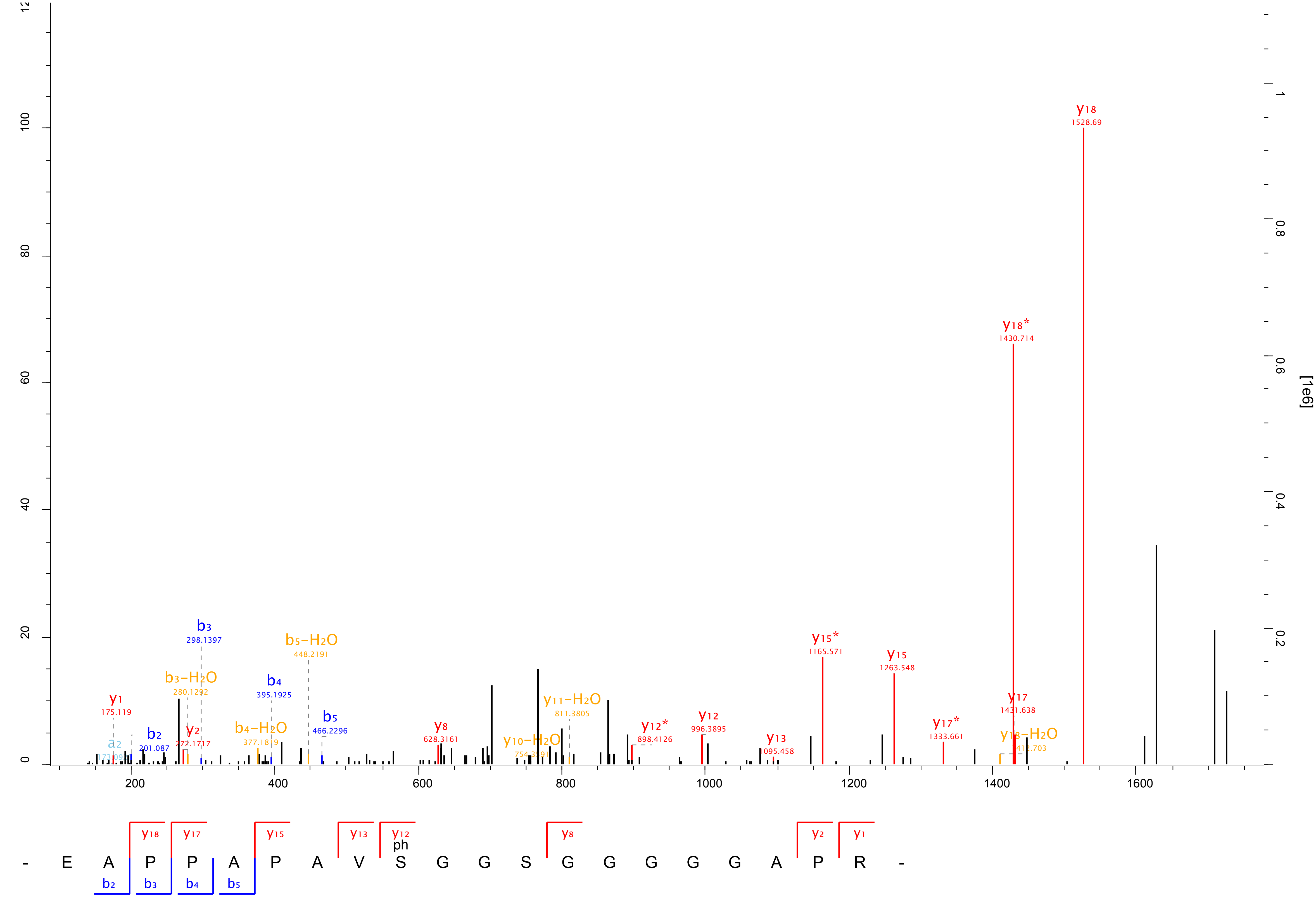

TraesCS5D01G132600.1 - S31

Raw File	Scan	Method	Score	m/z
E14720_1p_EXT-708_PSB_Inge_Ta_34C	14589	FTMS; HCD	191.33	570.27

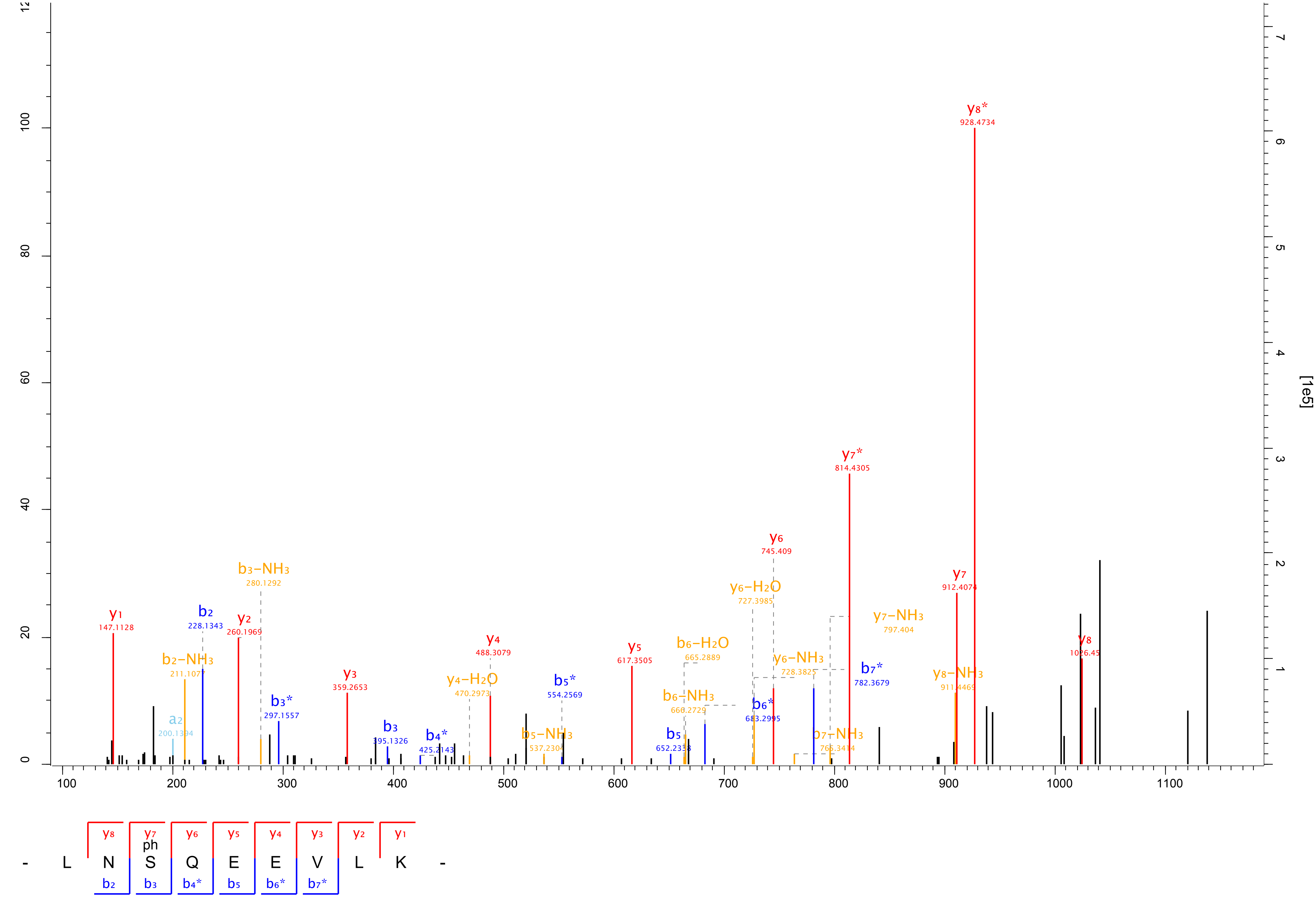

TraesCS4A01G328600.1 - S404

Raw File	Scan	Method	Score	m/z
E14716_1p_EXT-708_PSB_Inge_Ta_34A	9141	FTMS; HCD	163.33	845.8

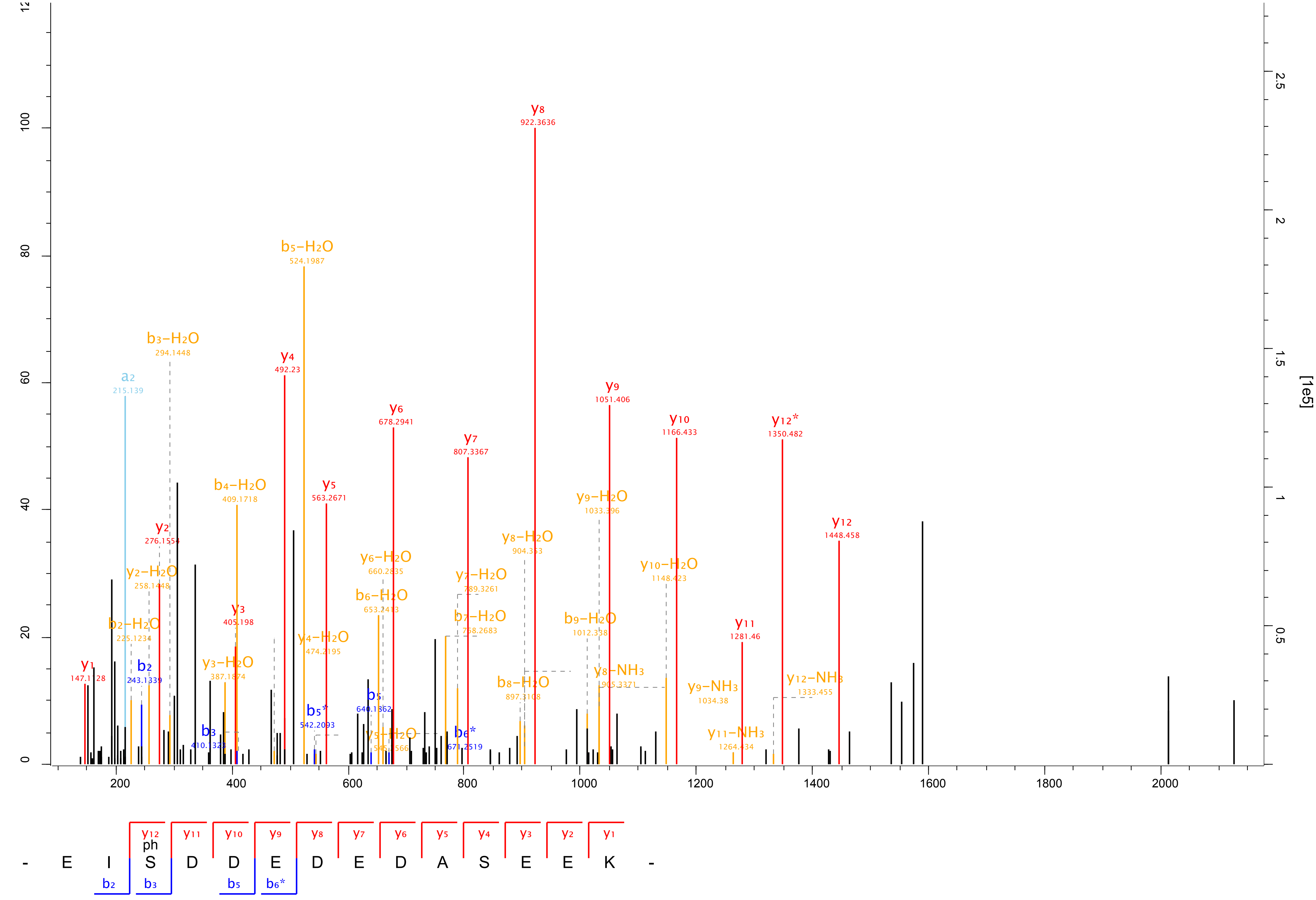

TraesCS2A01G033700.1 - S224

Raw File	Scan	Method	Score	m/z
E14720_1p_EXT-708_PSB_Inge_Ta_34C	20267	FTMS; HCD	97.73	590.27

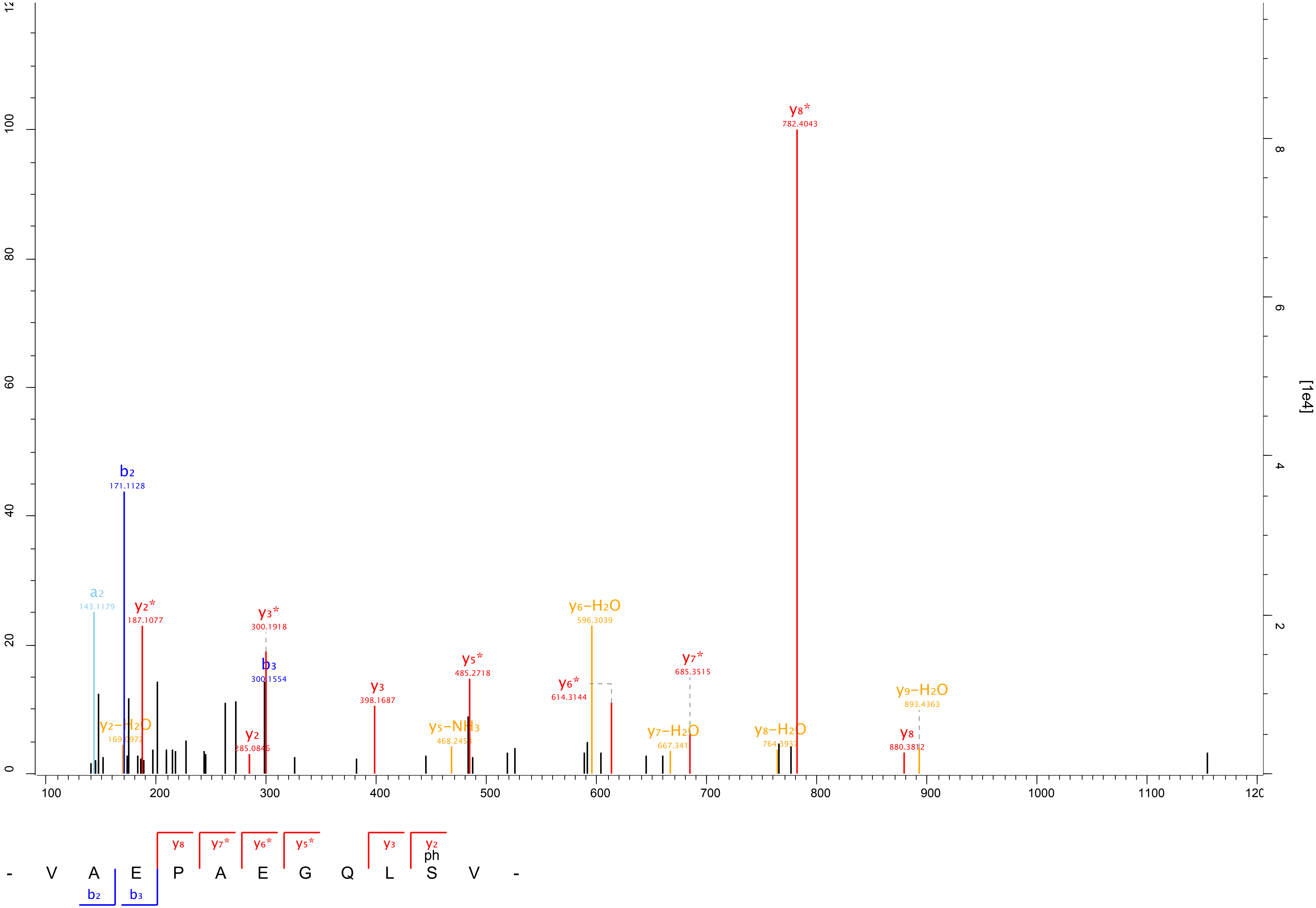

TraesCSU01G009200.1 - S577

Raw File	Scan	Method	Score	m/z
E16931_2p3_PSB-37_IVSME_tizhu_21-2	15559	FTMS; HCD	88.71	798.65

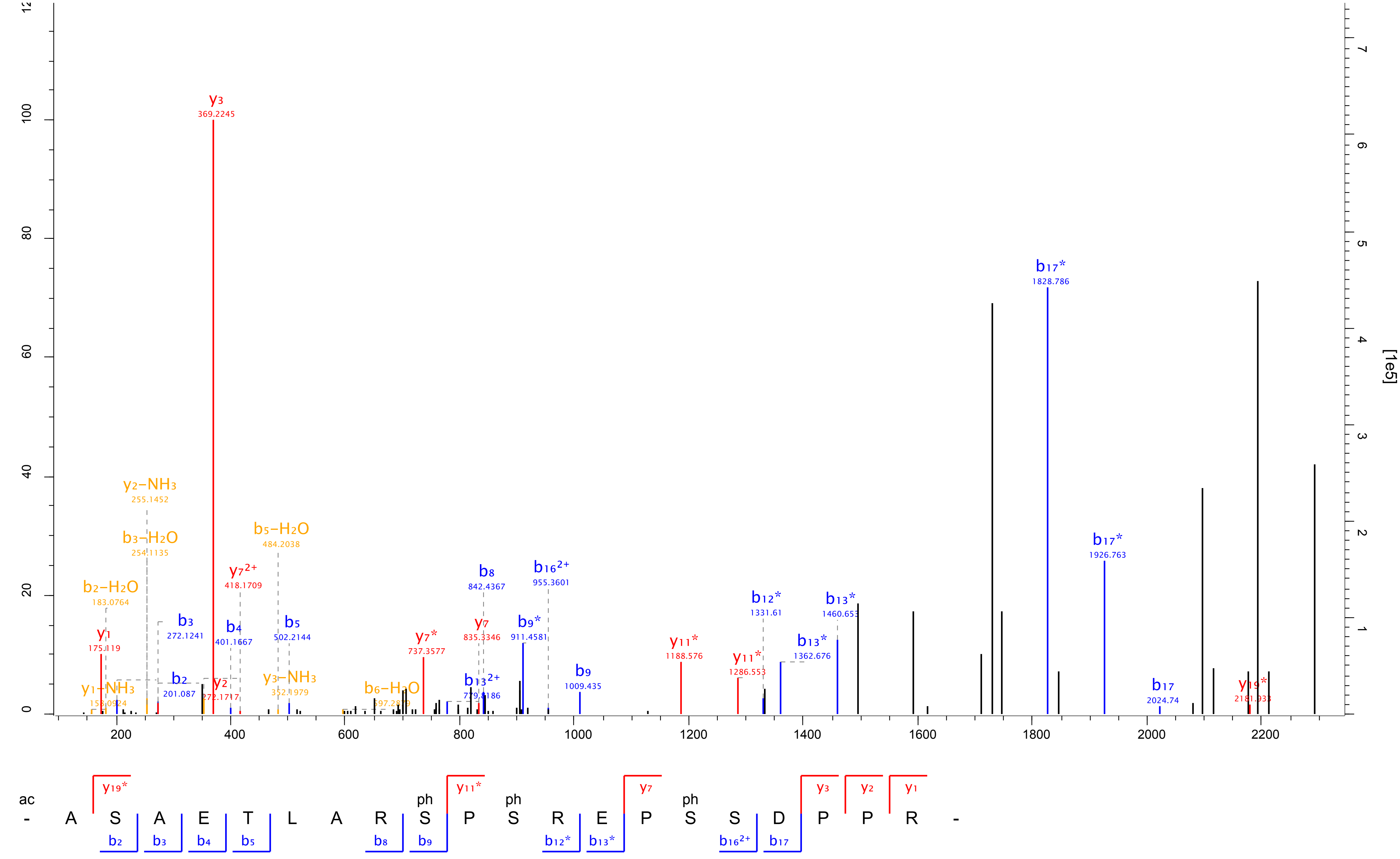

TraesCS2D01G281200.1 - S10 & S12

Raw File	Scan	Method	Score	m/z
E16939_2p3_PSB-37_IVSME_tizhu_34-3	14470	FTMS; HCD	111.66	783.86

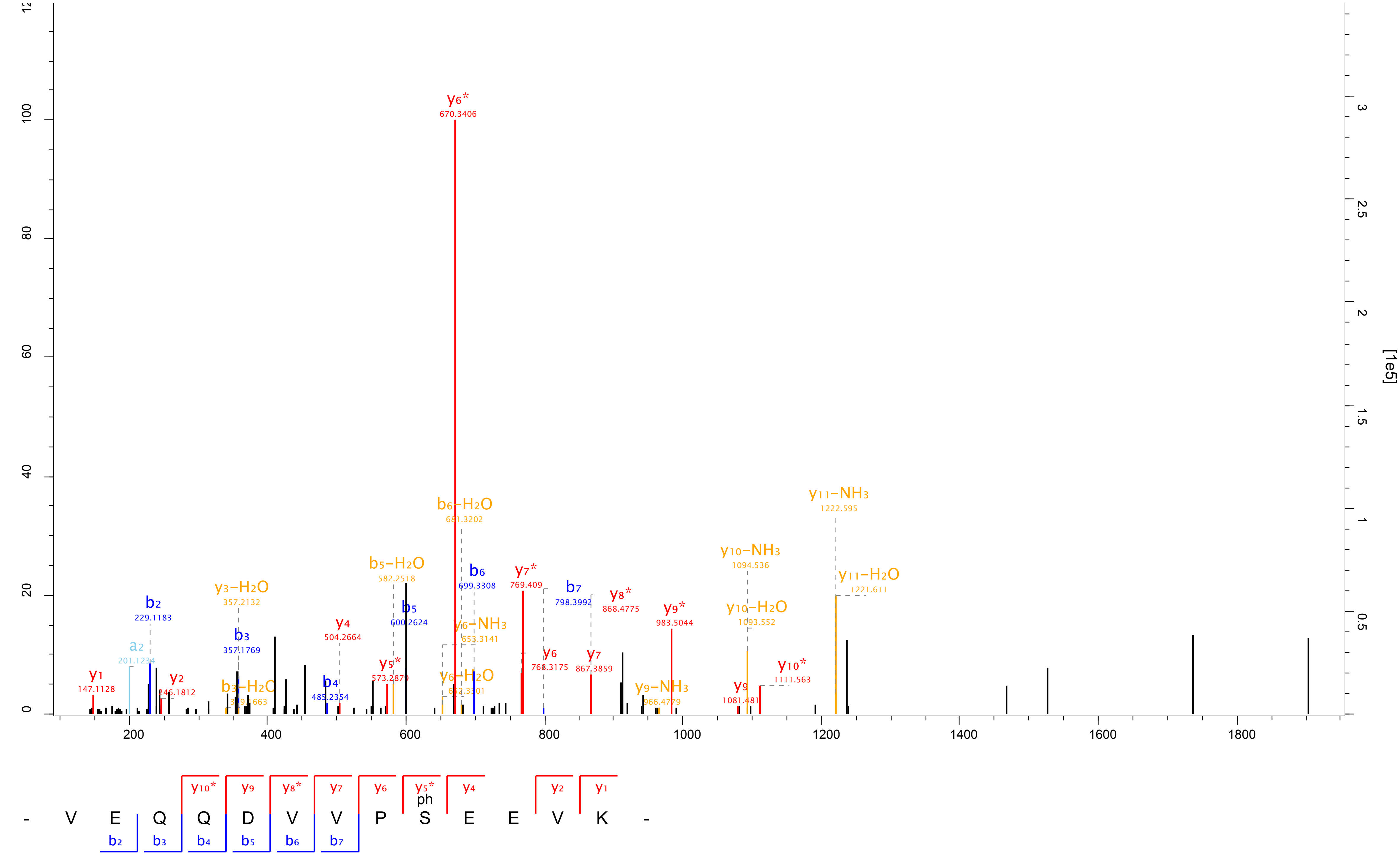

TraesCS2B01G177000.1 - S464

Raw File	Scan	Method	Score	m/z
E16939_2p3_PSB-37_IVSME_tizhu_34-3	20011	FTMS; HCD	115.05	704.8

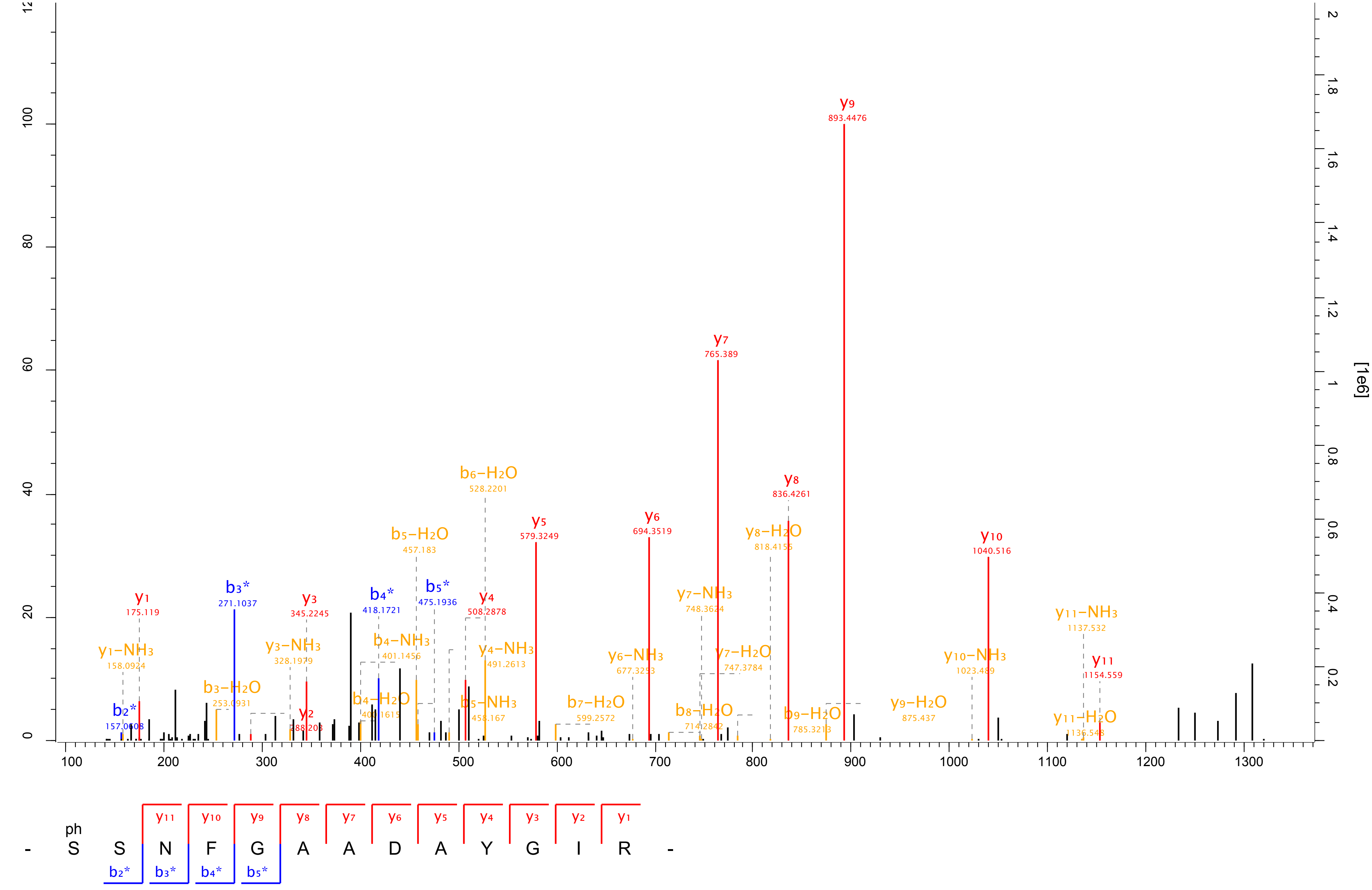

TraesCS6A01G308600.1 - S268&269

Raw File	Scan	Method	Score	m/z
E16929_2p3_PSB-37_IVSME_tizhu_21-1	13417	FTMS; HCD	92.41	706.97

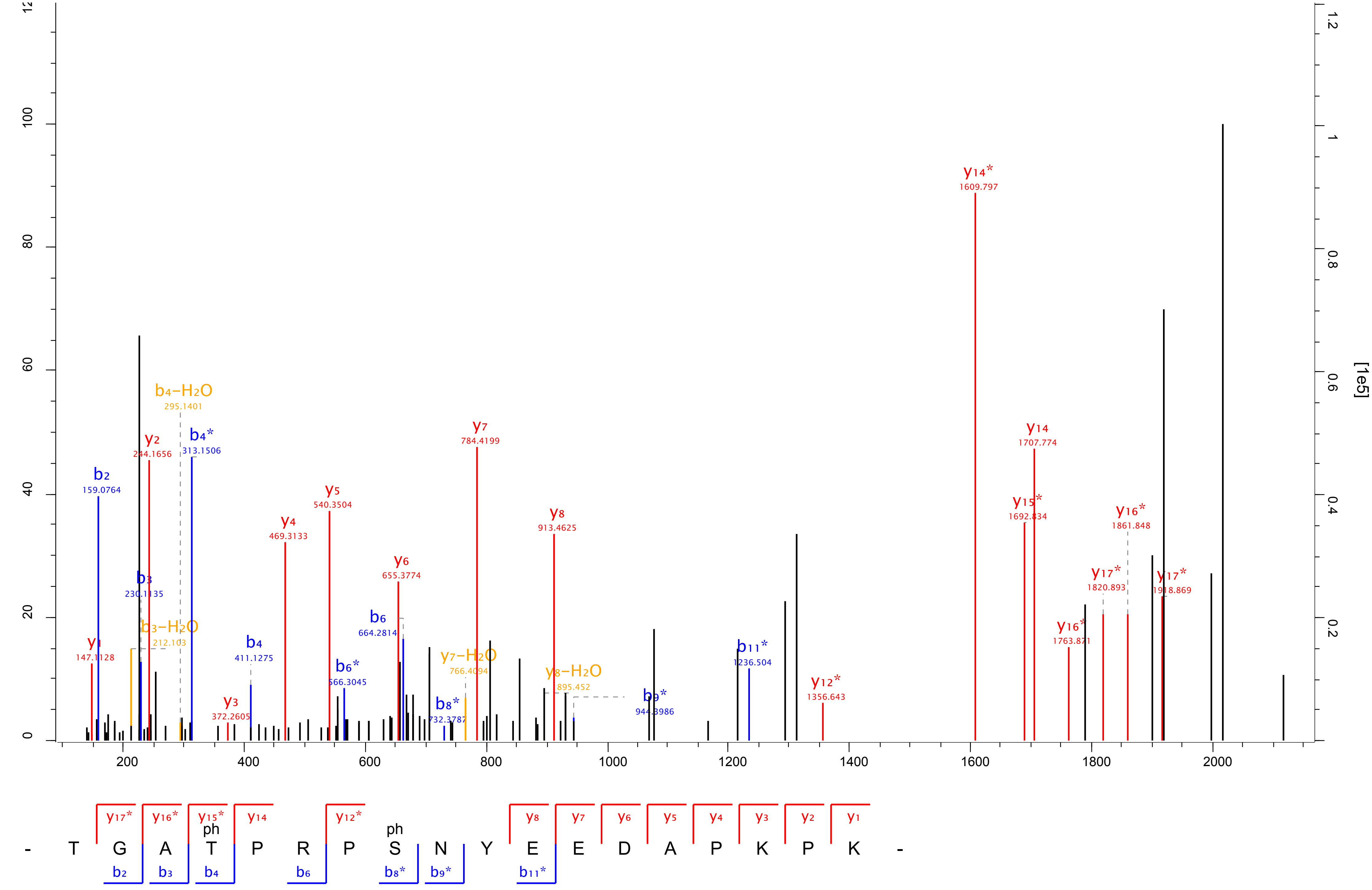

TraesCS6A01G308600.1 - T284

Raw File	Scan	Method	Score	m/z
E16929_2p3_PSB-37_IVSME_tizhu_21-1	11395	FTMS; HCD	121.95	714.81

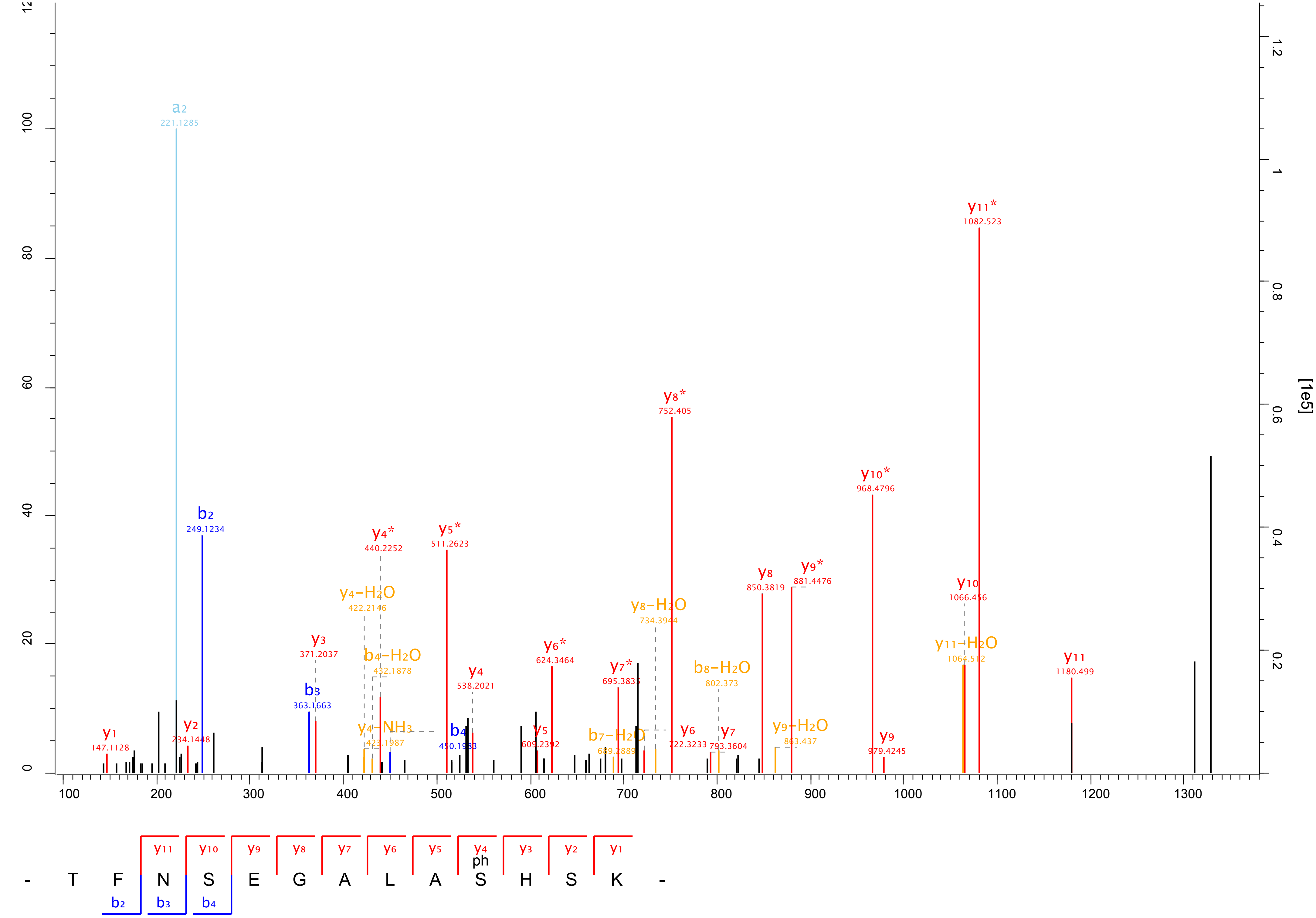

TraesCS1A01G445700.3 - S297

Raw File	Scan	Method	Score	m/z
E16935_2p3_PSB-37_IVSME_tizhu_34-1	26690	FTMS; HCD	84.04	1143.98

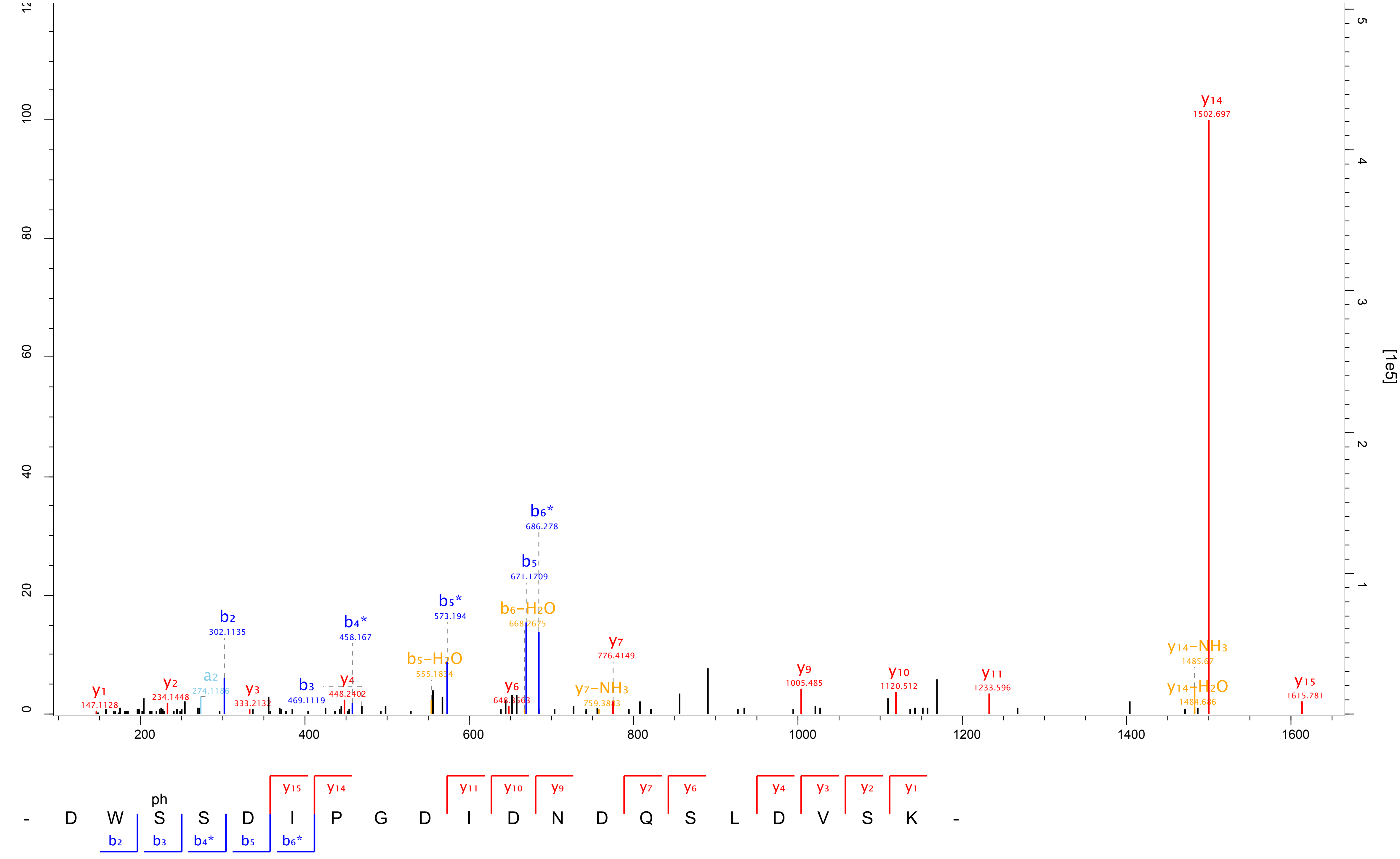

TraesCS2A01G262600.1 - S762

Raw File	Scan	Method	Score	m/z
E16935_2p3_PSB-37_IVSME_tizhu_34-1	11867	FTMS; HCD	67.64	554.94

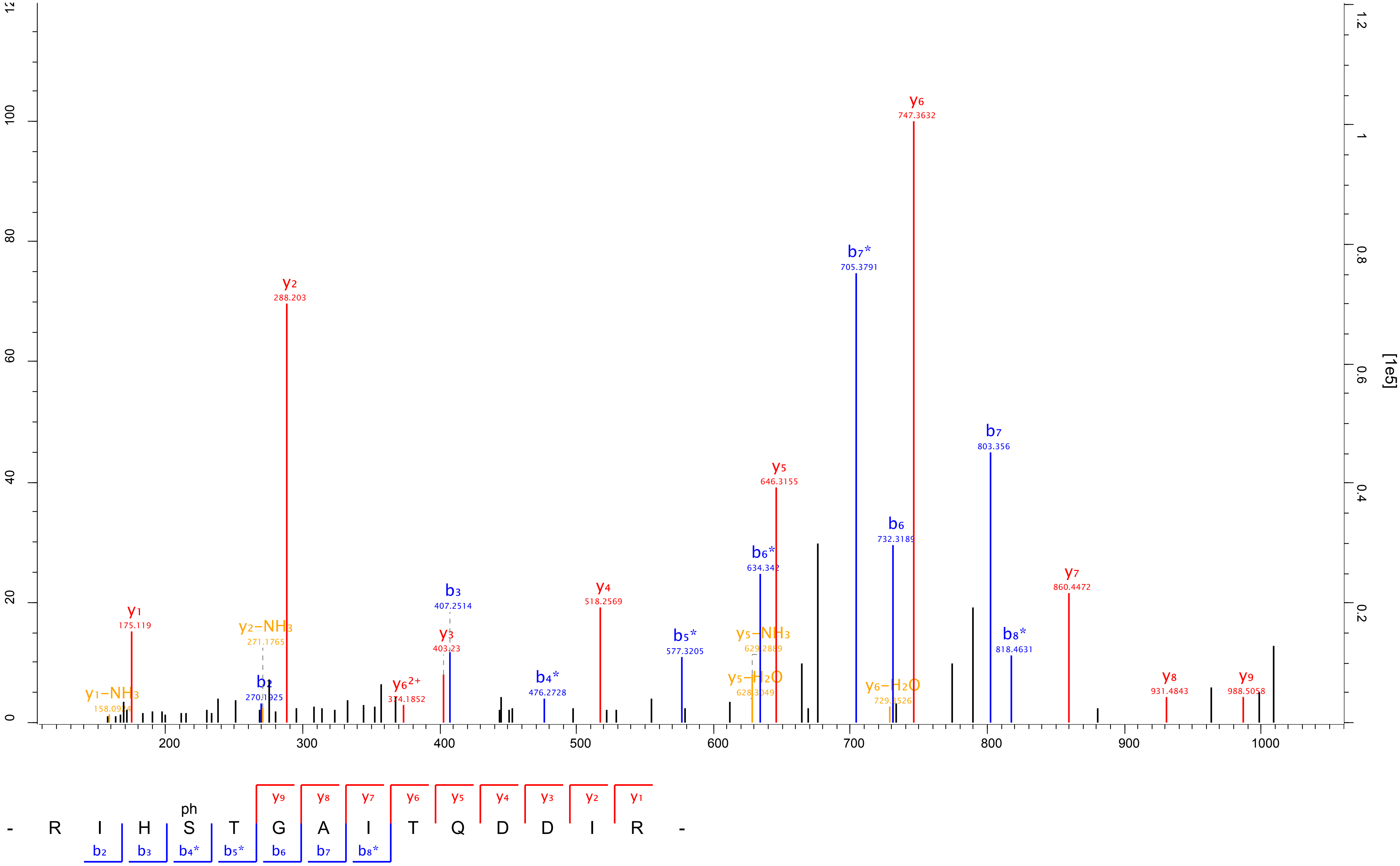

TraesCS4D01G266600.3 - S31

Raw File	Scan	Method	Score	m/z
E16939_2p3_PSB-37_IVSME_tizhu_34-3	11777	FTMS; HCD	54.76	554.94

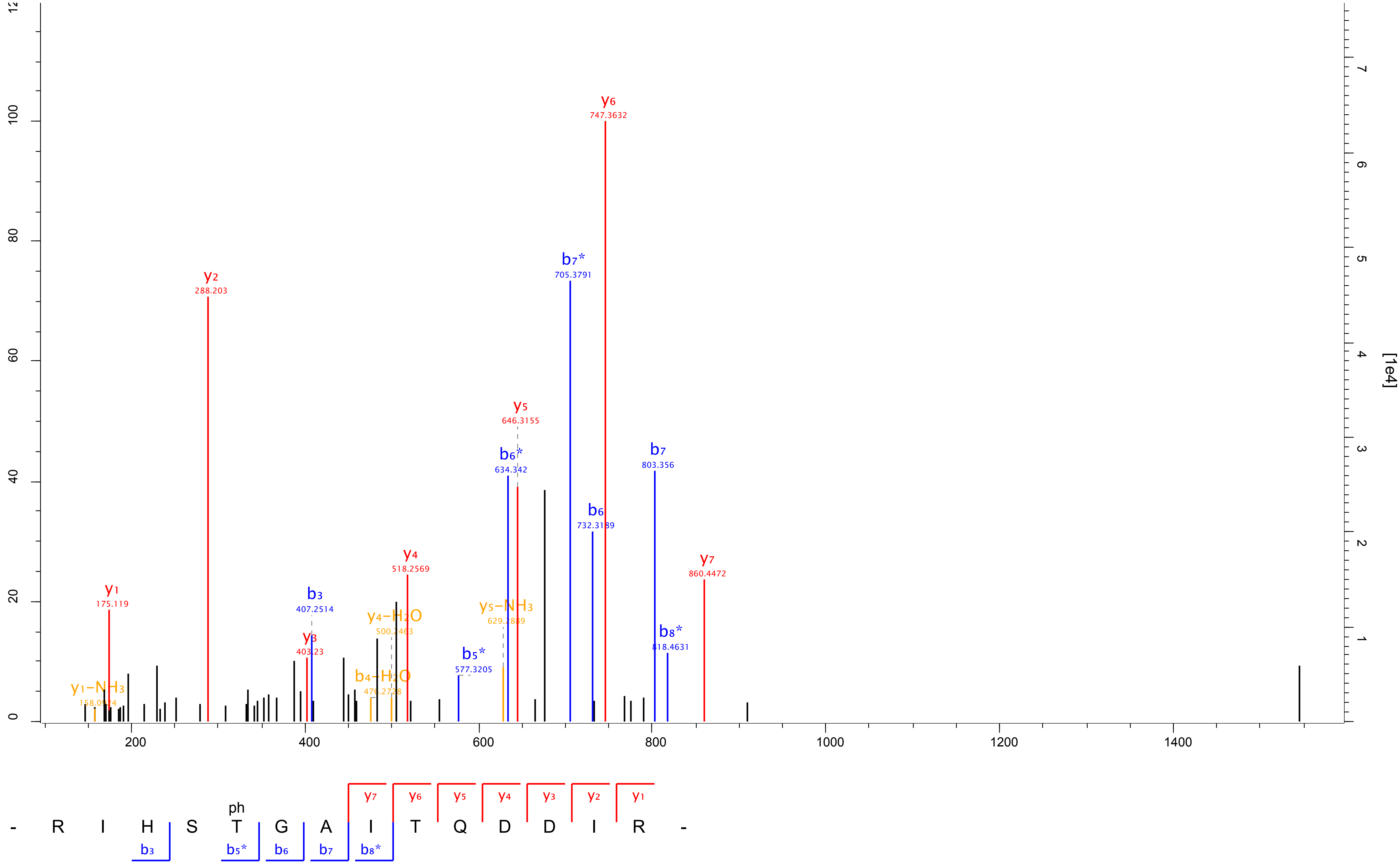

TraesCS4D01G266600.3 - T32

Raw File	Scan	Method	Score	m/z
E16939_2p3_PSB-37_IVSME_tizhu_34-3	28475	FTMS; HCD	42.11	909.43

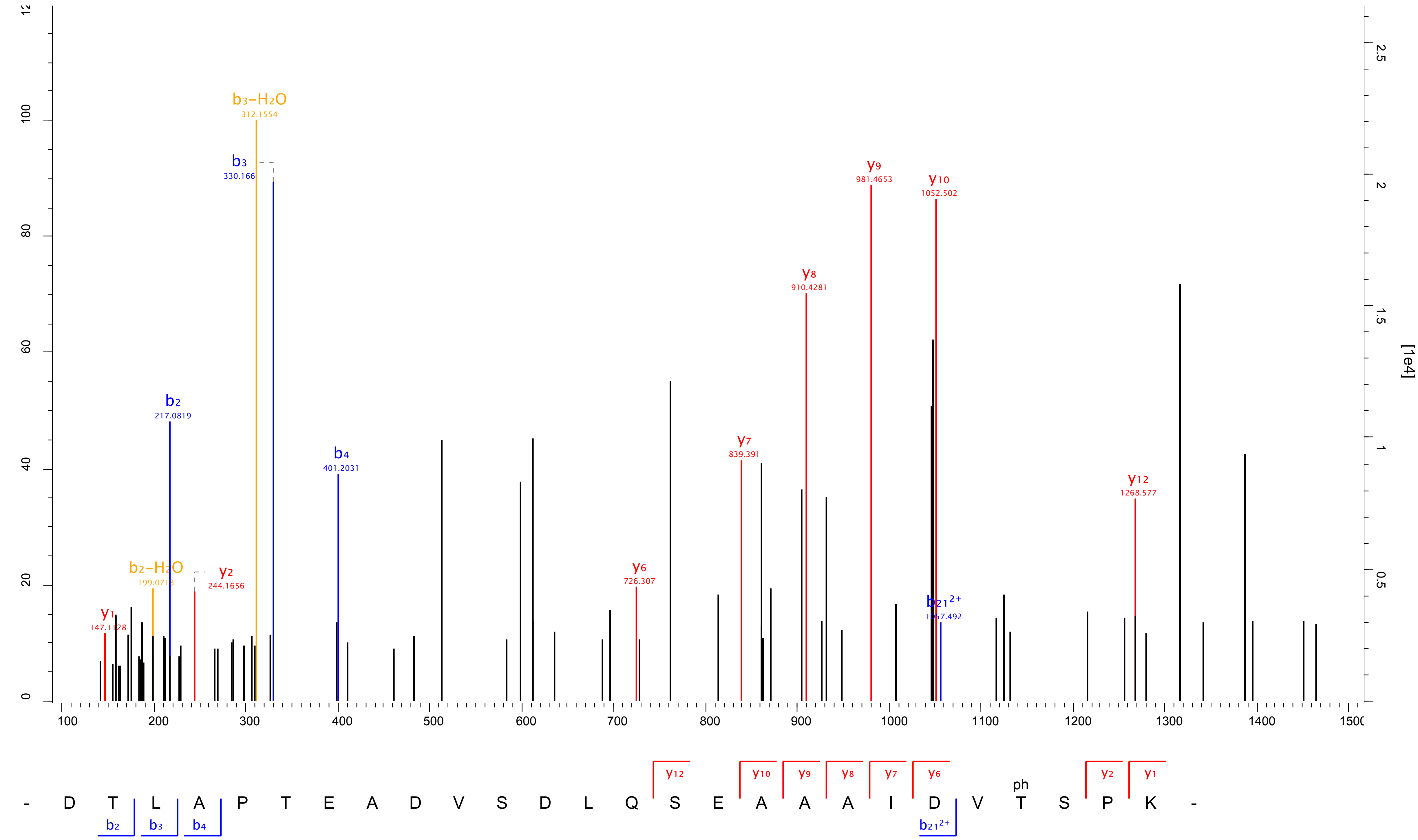

TraesCS7D01G206700.3 - T2492

Raw File	Scan	Method	Score	m/z
E16939_2p3_PSB-37_IVSME_tizhu_34-3	14139	FTMS; HCD	43.9	957.76

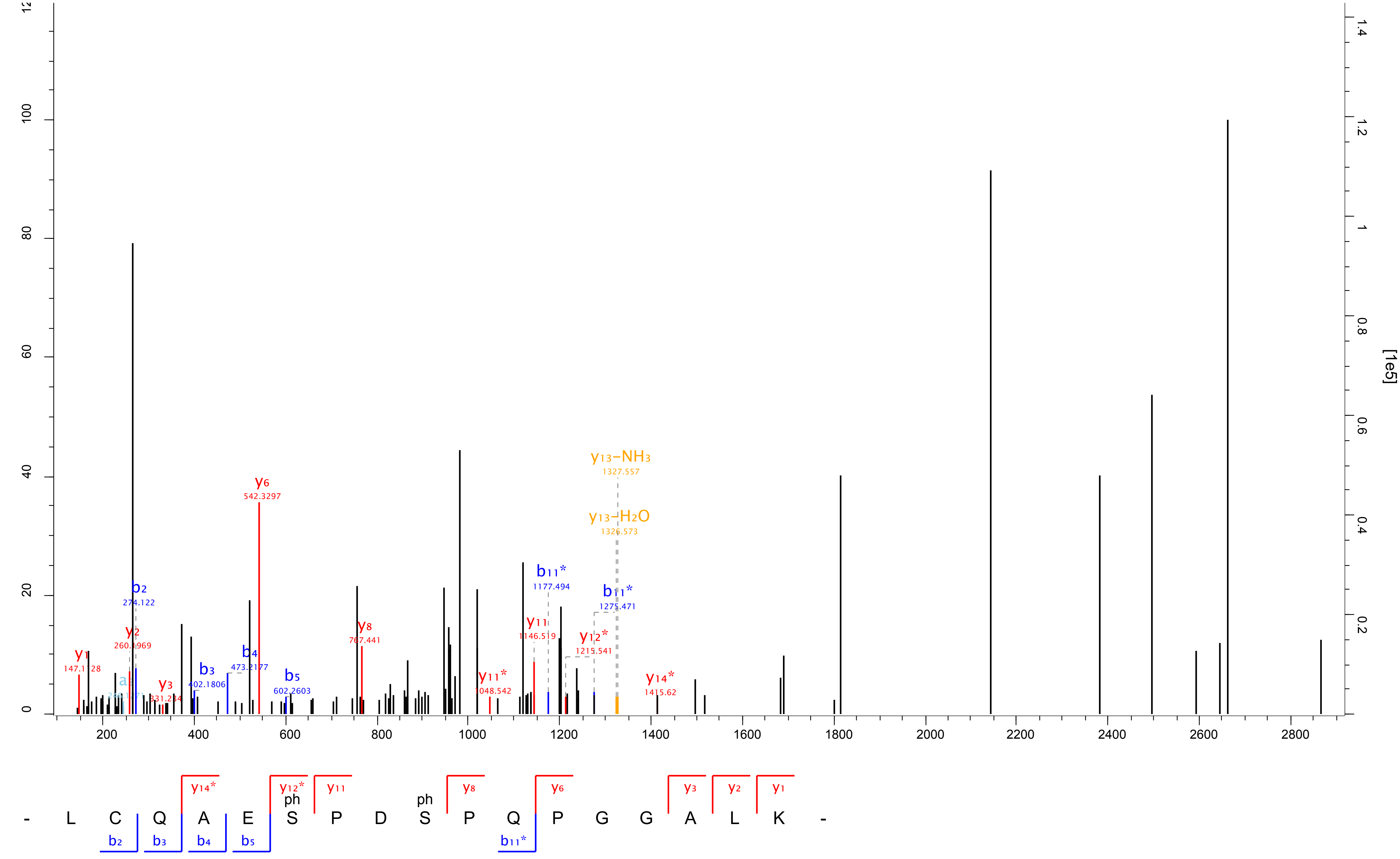

TraesCS7B01G110600.1 - S1668 and S1671

Raw File	Scan	Method	Score	m/z
E16931_2p3_PSB-37_IVSME_tizhu_21-2	17921	FTMS; HCD	59.34	605.64

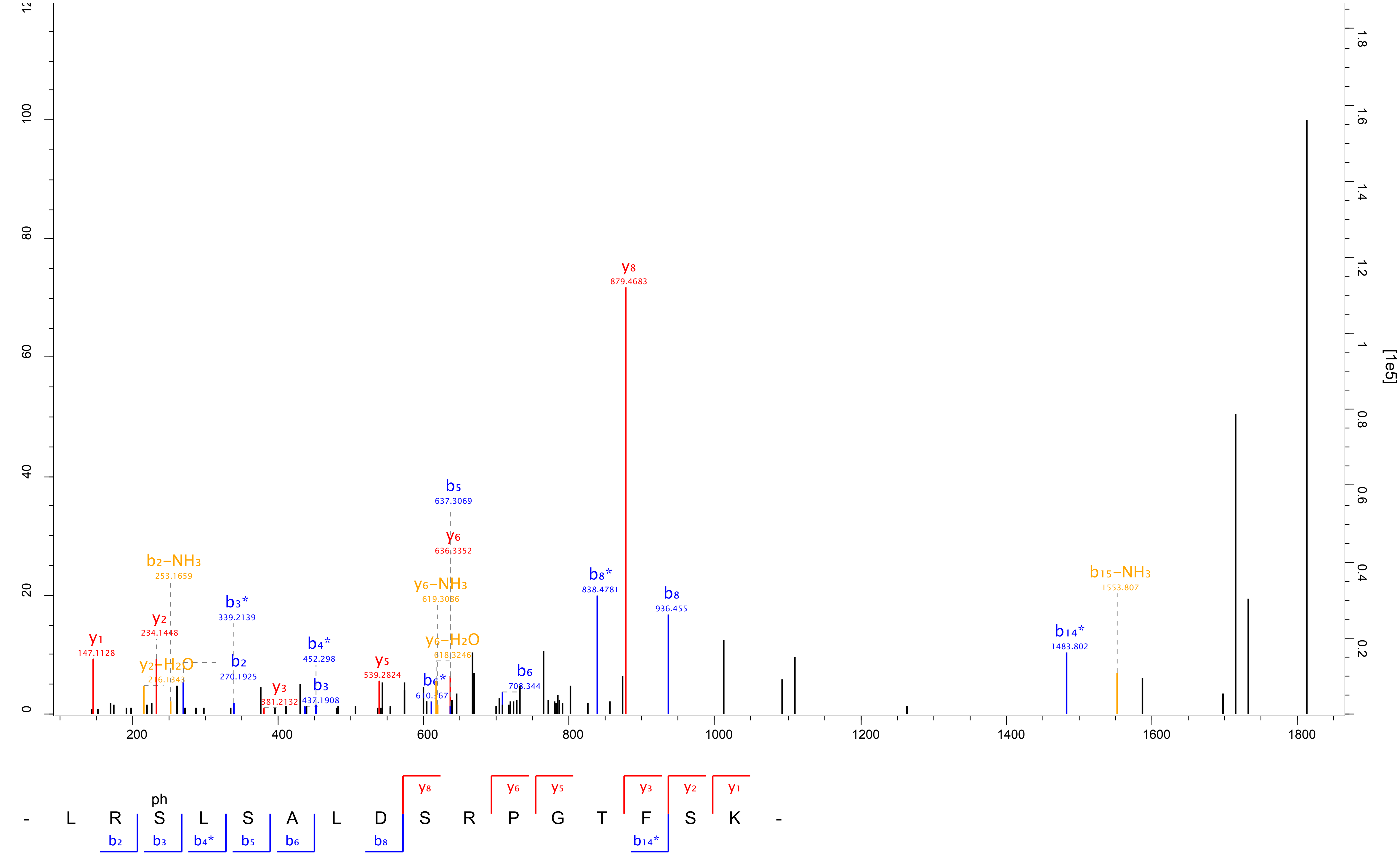

TraesCS6B01G048200.2 - S1728

Raw File	Scan	Method	Score	m/z
E16933_2p3_PSB-37_IVSME_tizhu_21-3	13385	FTMS; HCD	95.98	676.33

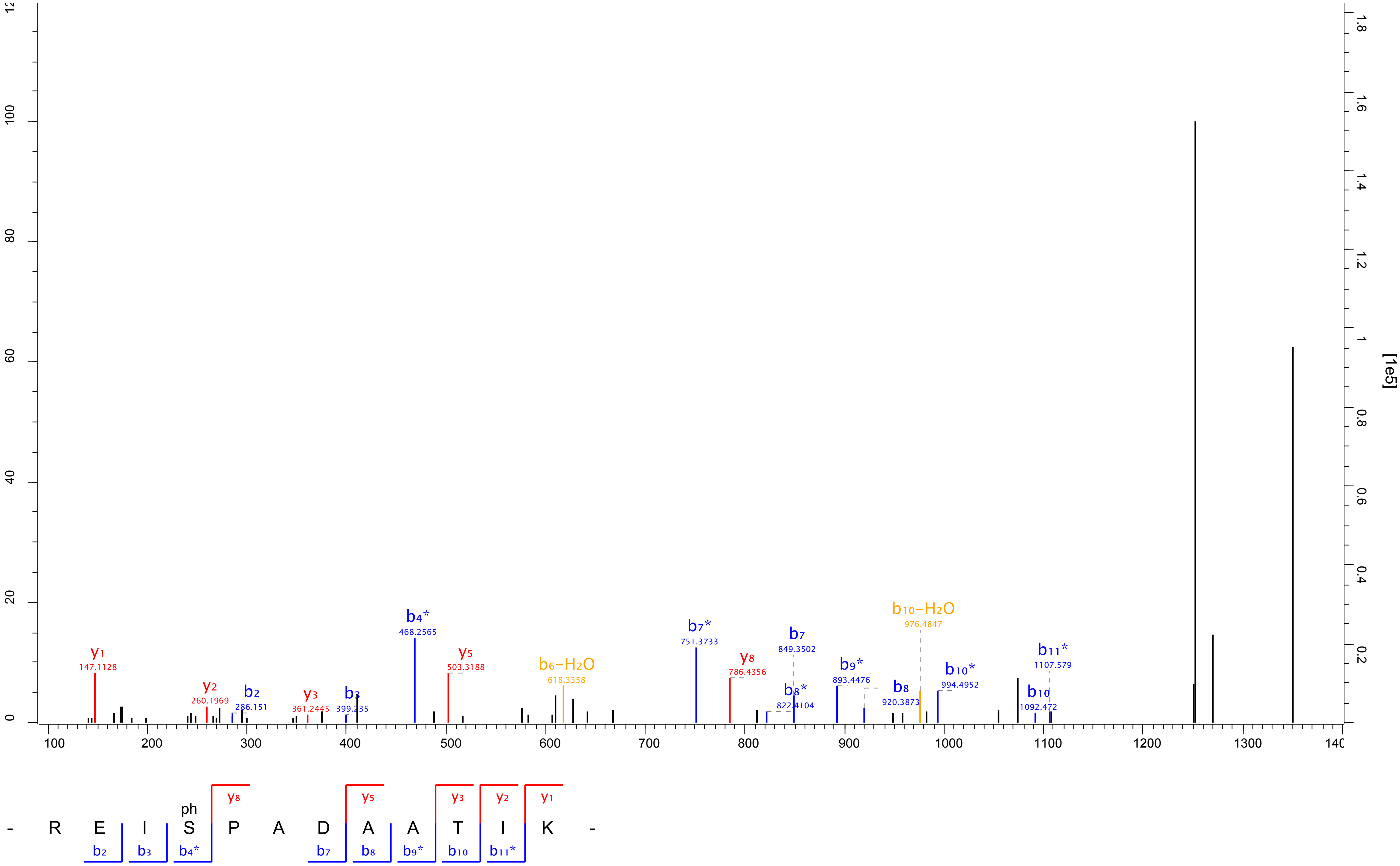

TraesCS5B01G410600.1 - S51

Raw File	Scan	Method	Score	m/z
E16939_2p3_PSB-37_IVSME_tizhu_34-3	30572	FTMS; HCD	48.66	705.37

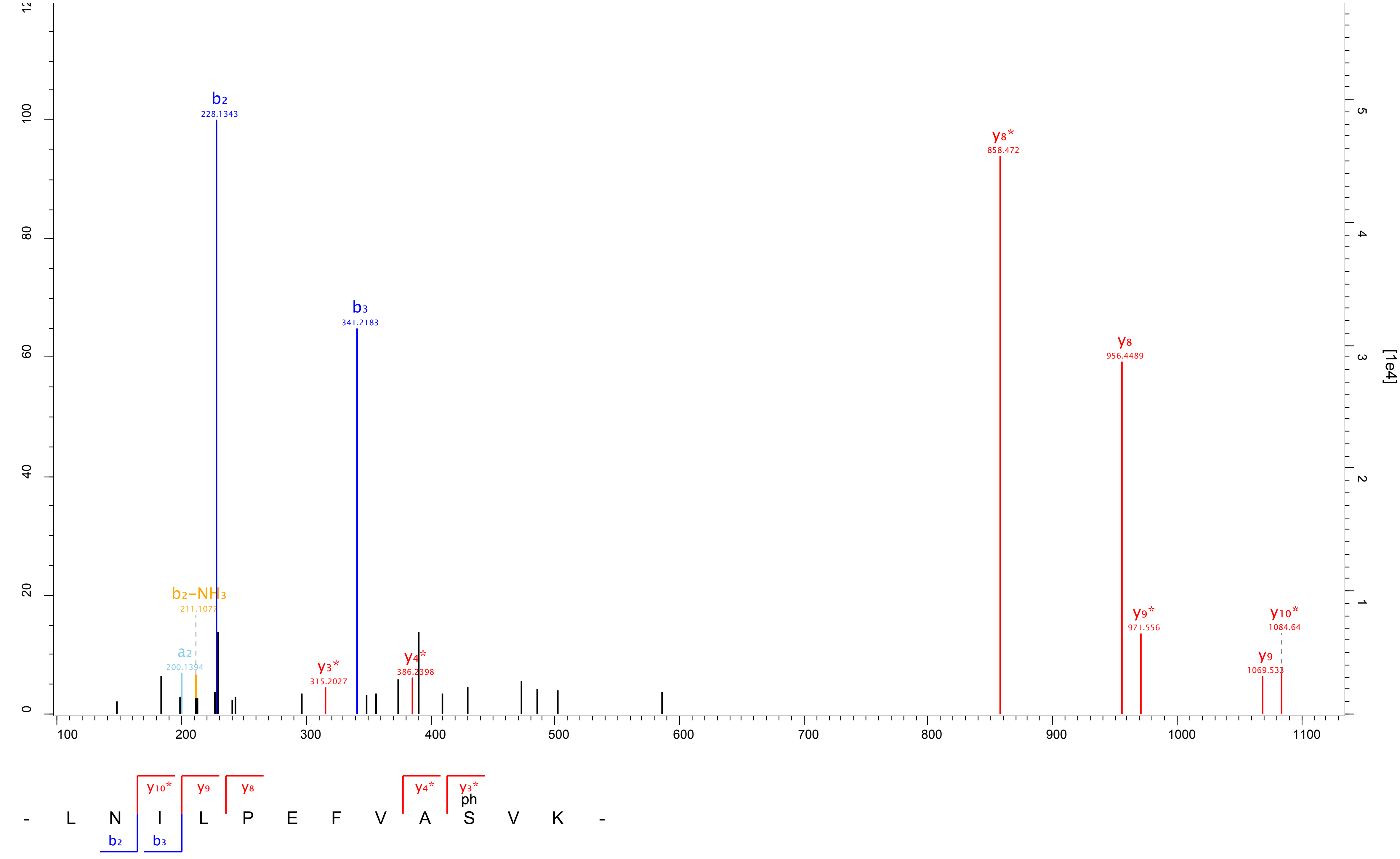

TraesCS6B01G377500.3 - S711

Raw File	Scan	Method	Score	m/z
E16939_2p3_PSB-37_IVSME_tizhu_34-3	10732	FTMS; HCD	117.57	889.69

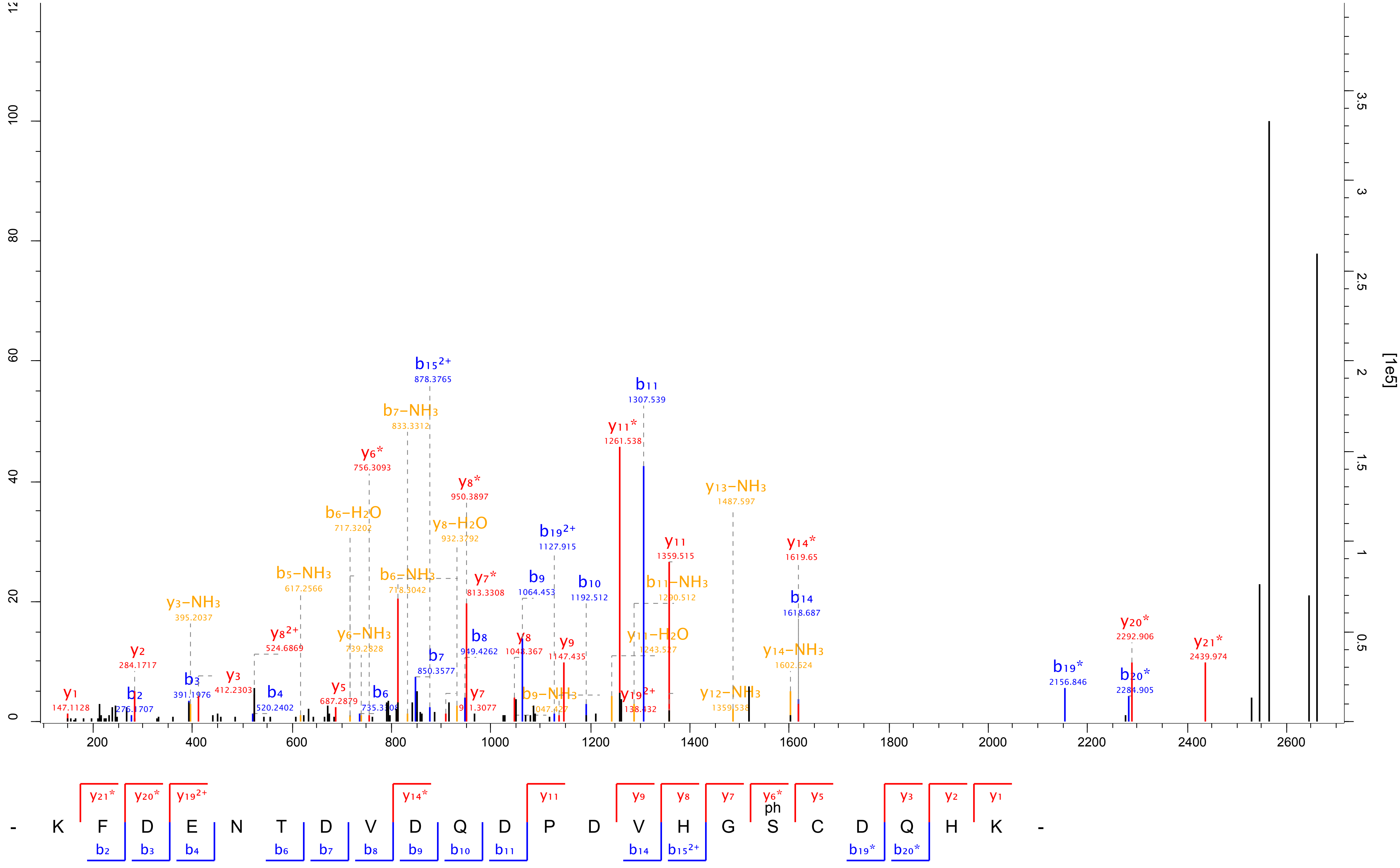

TraesCS6B01G377500.3 - S762

Raw File	Scan	Method	Score	m/z
E16937_2p3_PSB-37_IVSME_tizhu_34-2	25277	FTMS; HCD	122.44	1068.49

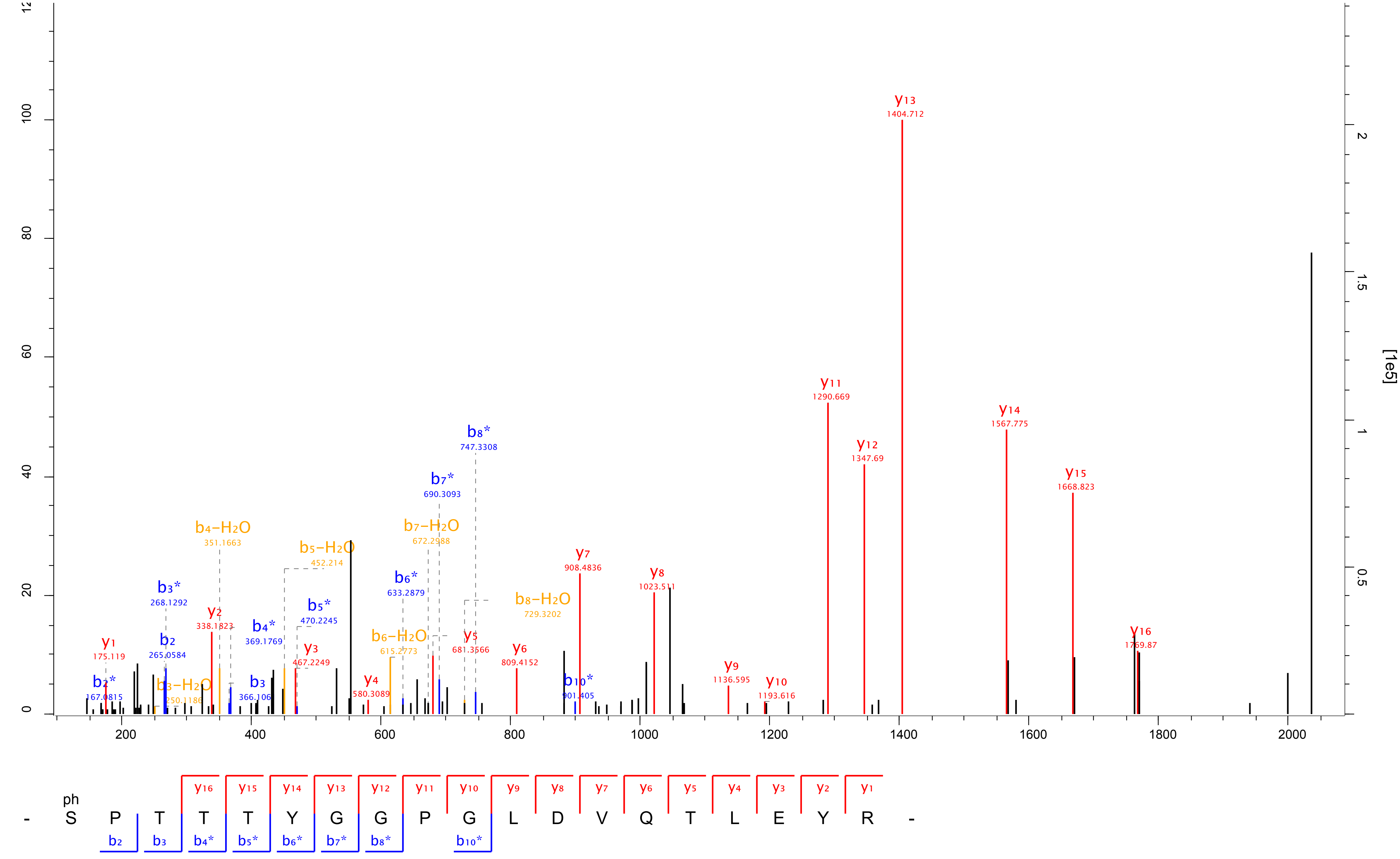

TraesCS5B01G387800.1 - S3236

Raw File	Scan	Method	Score	m/z
E16929_2p3_PSB-37_IVSME_tizhu_21-1	25242	FTMS; HCD	171.87	1068.49

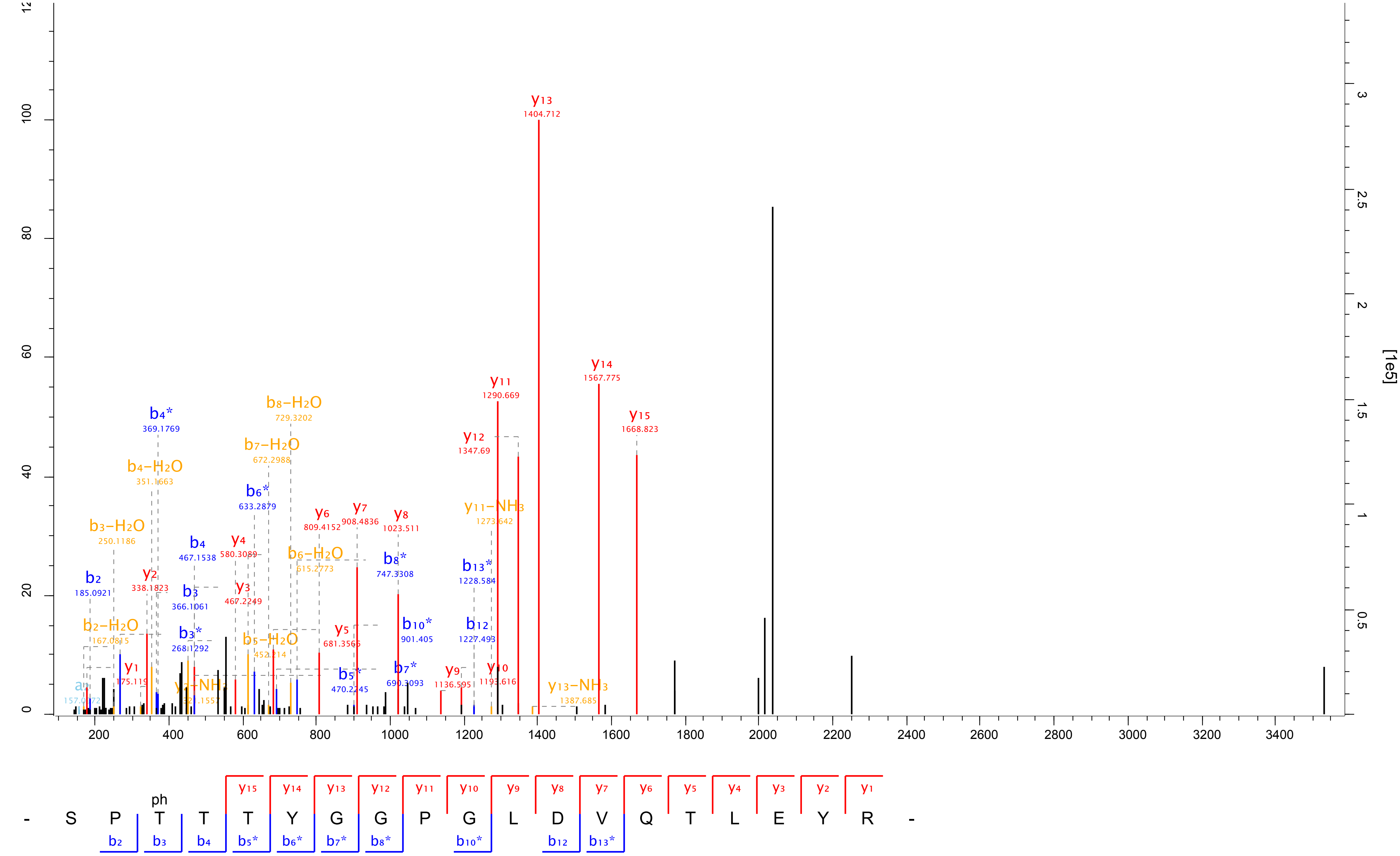

TraesCS5B01G387800.1 - T3238

Raw File	Scan	Method	Score	m/z
E16939_2p3_PSB-37_IVSME_tizhu_34-3	14710	FTMS; HCD	94.59	772

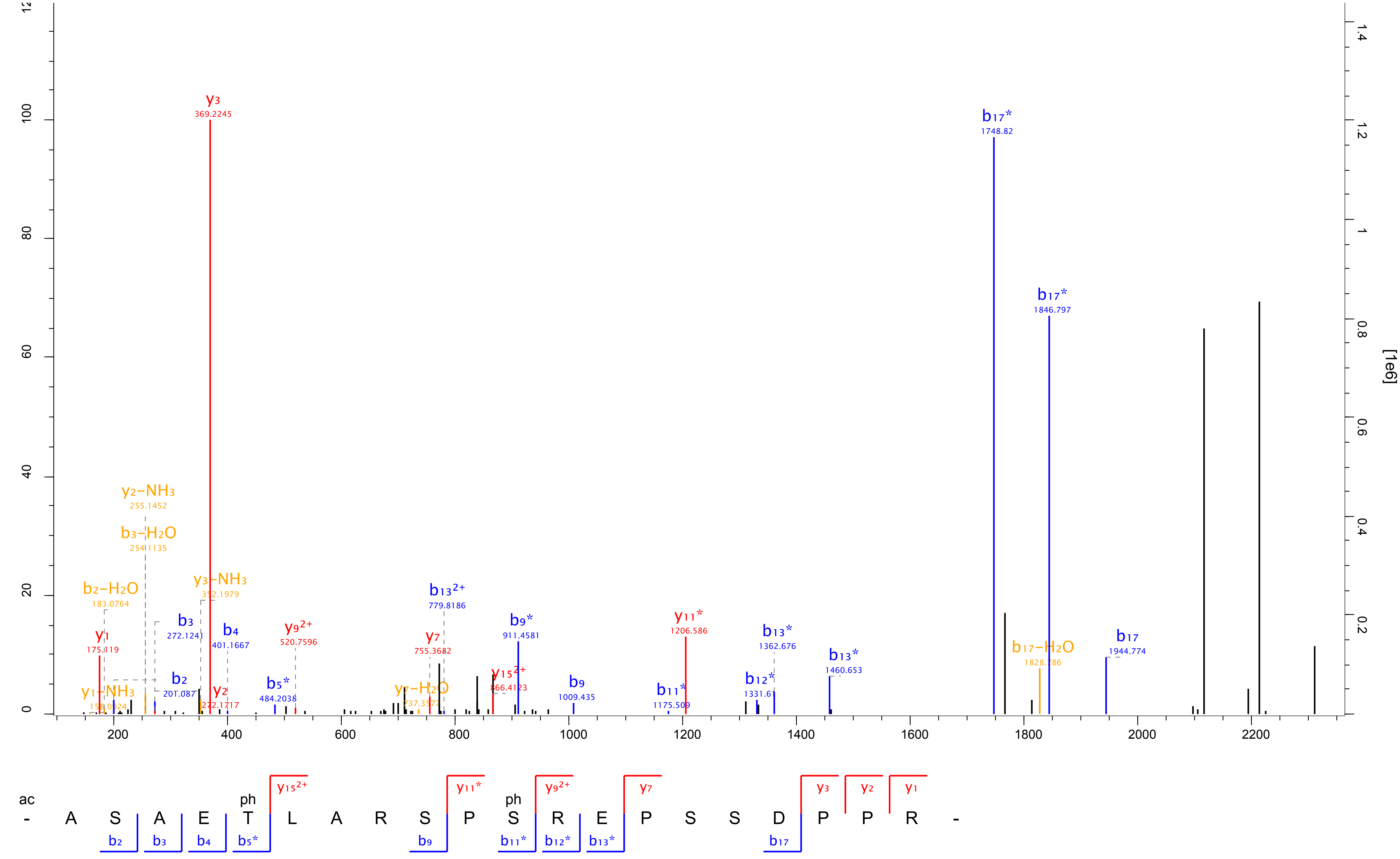

TraesCS2D01G281200.1 - S12
